# IMiD/CELMoD-induced growth suppression of adult T-cell leukemia/lymphoma cells *via* cereblon through downregulation of target proteins and their downstream effectors

**DOI:** 10.3389/fonc.2023.1272528

**Published:** 2024-01-24

**Authors:** Yu Wang, Shunsuke Shimosaki, Emi Ikebe, Hidekatsu Iha, Jun-ichi Yamamoto, Nichole Fife, Tomonaga Ichikawa, Mitsuo Hori, Masao Ogata, Yoshiyuki Tsukamoto, Naoki Hijiya, Masatsugu Moriyama, Shotaro Hagiwara, Shuichi Kusano, Masumichi Saito, Kamruddin Ahmed, Akira Nishizono, Hiroshi Handa, Kazuhiro Morishita

**Affiliations:** ^1^ Department of Microbiology, Faculty of Medicine, Oita University, Yufu, Japan; ^2^ Division of Tumor and Cellular Biochemistry, Department of Medical Sciences, Faculty of Medicine, University of Miyazaki, Miyazaki, Japan; ^3^ Research Center for Biological Products in the Next Generation, National Institute of Infectious Diseases, Musashi-murayama, Japan; ^4^ Division of Pathophysiology, The Research Center for GLOBAL and LOCAL Infectious Diseases (RCGLID), Oita University, Yufu, Japan; ^5^ Borneo Medical and Health Research Centre, Faculty of Medicine and Health Sciences, University of Malaysia Sabah, Kota Kinabalu, Malaysia; ^6^ Department of Nanoparticle Translational Research, Tokyo Medical University, Tokyo, Japan; ^7^ Department of Hematology, Ibaraki Prefectural Central Hospital, Kasama, Japan; ^8^ Department of Hematology and Oncology, Faculty of Medicine, Oita University, Yufu, Japan; ^9^ Department of Molecular Pathology, Faculty of Medicine, Oita University, Yufu, Japan; ^10^ Mito Center for Regional Medical Education, University of Tsukuba, Ibaraki, Japan; ^11^ Division of Biological Information Technology, Joint Research Center for Human Retrovirus Infection, Graduate School of Medical and Dental Sciences, Kagoshima University, Kagoshima, Japan

**Keywords:** adult T-cell lymphoma/leukemia, HTLV-1, immunomodulatory drug (IMiDs), cereblon modulator (CELMoD), IRF4, IKZF2, CRBN

## Abstract

Adult T-cell leukemia/lymphoma (ATL) is an aggressive T-cell neoplasia associated with human T-cell leukemia virus type 1 (HTLV-1) infection and has an extremely poor prognosis. Lenalidomide (LEN; a second-generation immunomodulatory drug [IMiD]) has been employed as an additional therapeutic option for ATL since 2017, but its mechanism of action has not been fully proven, and recent studies reported emerging concerns about the development of second primary malignancies in patients treated with long-term IMiD therapy. Our purpose in this study was to elucidate the IMiD-mediated anti-ATL mechanisms. Thirteen ATL-related cell lines were divided into LEN-sensitive or LEN-resistant groups. *CRBN* knockdown (KD) led to a loss of LEN efficacy and *IKZF2*-KD-induced LEN efficacy in resistant cells. DNA microarray analysis demonstrated distinct transcriptional alteration after LEN treatment between LEN-sensitive and LEN-resistant ATL cell lines. Oral treatment of LEN for ATL cell-transplanted severe combined immunodeficiency (SCID) mice also indicated clear suppressive effects on tumor growth. Finally, a novel cereblon modulator (CELMoD), iberdomide (IBE), exhibited a broader and deeper spectrum of growth suppression to ATL cells with efficient IKZF2 degradation, which was not observed in other IMiD treatments. Based on these findings, our study strongly supports the novel therapeutic advantages of IBE against aggressive and relapsed ATL.

## 1 Introduction

ATL is an aggressive malignancy of peripheral T lymphocytes caused by human T-cell leukemia virus type 1 (HTLV-1), and its prognosis is extremely poor compared to that of other non-Hodgkin lymphomas (1). HTLV-1 is transmitted through sexual intercourse, blood transfusions, and mother-to-child breastfeeding, which are crucial in developing ATL (2). The cumulative risks for ATL development among HTLV-1 carriers are estimated at approximately 5% (1, 2). The initial expansion of HTLV-I infected cells is driven by the viral oncoprotein Tax, which activates transcription of the proviral genome from the proviral promoter and induces T-cell autocrine loops of IL-2, IL-15, and their respective receptors through Tax–I-κBα kinase axis (3). Tax alters functions of many transcription factors including CREB/ATF, AP-1, and NF-κB to upregulate growth as well as anti-apoptotic signals and represses p53, DNA polymerase beta, PCNA, and MAD-1 to interfere with multiple cell cycle regulatory and DNA repair systems (3). Another viral oncoprotein, HBZ, encoded by the complementary strand of HTLV-1 provirus genome, also interacts with multiple transcription factors, such as Sp1, Maf, and JunD, to promote cellular proliferation and induce transcription of Foxp3, CCR4, and TIGIT to evade host cellular immune response to HTLV-1-infected cells and eventually promotes ATL onset (3).

Because of randomized host-genomic integration of HTLV-1 provirus, ATL is a genetically diverse condition, which is manifested by variations in clinical outcome and is classified into four subtypes (acute, lymphoma, chronic, and smoldering), which display distinct clinical features and therapeutic responses, thus requiring multiplex clinical management. In the cases of aggressive ATL (acute, lymphoma, or unfavorable chronic types), intensive chemotherapy followed by allogeneic hematopoietic stem cell transplantation is the standard procedure, and for indolent ATL (favorable chronic or smoldering types), watchful waiting until disease progression has been recommended in Japan (1, 2). As a whole, current treatments of aggressive ATL are dismal, and refractory or relapsed ATL is highly resistant to salvage therapy (1). Since most ATL cells express a chemokine receptor CCR4, expressed intrinsically in regulatory T cell (Treg), in their cellular surfaces through the HBZ-FoxP3 pathway, a humanized monoclonal anti-CCR4 antibody mogamulizumab is a novel and promising therapeutic agent for ATL (4). Even though classic and novel therapies are applicable to ATL, its multifaceted pathogenic features urgently require additional therapeutic options.

IRF4 is one of the most activated/amplified genes in the genome of aggressive ATL patient subtypes (5, 6). Since IRF4 knockdown induces apoptotic profiles in ATL cells, IRF4 seems to play a crucial role in ATL development. IRF4, coordinately with NF-κB, regulates multiple cancer-related gene expressions such as MYC, SMAD2, and IKZF2 (6). The expression of IRF4 is mainly regulated by IKZF1 and IKZF3 (7, 8); thus, IKZF1/3 could also play a crucial role in ATL development.

Lenalidomide (LEN) is characterized as an IMiD compound and has been approved for hematological malignancies such as multiple myeloma (MM) (9) and ATL (2). LEN is known to modulate CRBN substrate specificity, and CRBN attaches ubiquitin to multiple targets to lead them into proteasomal degradation (8). With this functional modulation, LEN significantly downregulates IKZF1/3 and exerts its therapeutic action on the patients of MM and 5q-deletion myelodysplastic syndrome (5q-MDS) (9, 10). Although there have been two successful multicenter phase 1/2 clinical studies of LEN efficacy for ATL patients having relapse or recurrence from the initial treatment (11, 12) or effective maintenance therapy for aggressive ATL (13), the precise mechanisms of LEN efficacy against ATL cells are still elusive. In this study, we evaluated the effects of LEN and other IMiDs against ATL cells with both *in vitro* and *in vivo* ATL preclinical models to elucidate the direct cellular growth inhibition effects on ATL cells.

## 2 Materials and methods

### 2.1 Cell lines and cultures

The characteristics of each cell line are summarized in **Supplementary Material 1**. Each cell line was maintained using RPMI-1640 culture media supplemented with 15% fetal calf serum (FCS, St. Louis, MO, USA), penicillin G (50 U/mL), and streptomycin (50 μg/mL). Antibiotics were purchased from Sigma-Aldrich Co., LLC (St. Louis, MO, USA).

### 2.2 IMiDs, CELMoD, and cell treatment

IMiDs [LEN and pomalidomide (POM)] and cereblon modulator (CELMoD) (iberdomide (IBE) were purchased from Selleck (Tokyo, Japan). These chemicals were initially dissolved in dimethyl sulfoxide (DMSO) at 200 mM as stock solutions, diluted with DMSO for each concentration immediately before use, and then added to cultured media at 1/200 volume. Half of the culture media and cell suspensions were replaced with fresh media on days 3 and 5 immediately after the cell viability assay.

### 2.3 Cell viability assay

Cells were plated in each 24-well at 5 × 10^4^ cells per 1.5 mL RPMI-1640 and kept in a 5% CO_2_ incubator for 24 hours (cell number should reach 1 × 10^5^). Before (day 0) or after (days 1, 2, and 3) the addition of lenalidomide (7.5 µL, 200× concentration each) to the cultured media, approximately 800 cells in 12.5 μL media were harvested and mixed with an equal volume of CellTiter solution (Promega, Madison, WI, USA) for each assay (triplicated). After 10 minutes of incubation at room temperature, the reaction mixture in a 96-well plate was read using a GloMax luminometer (Promega, Tokyo, Japan).

### 2.4 Western blotting analysis

Three to five million cells were prepared and lysed with HEPES (pH 7.3)/NP40 (0.5%) cell lysis buffer supplemented with protease inhibitor mix (Roche, Basel, Switzerland). Antibodies used in this report are summarized in **Supplementary Material 2**.

### 2.5 Real-time PCR analysis

Cells with or without lenalidomide treatment were harvested, and total RNAs were prepared using the RNeasy kit (Qiagen, Tokyo, Japan) for RT-PCR assay. Universal Probe Library and Light Cycler 480 system were employed for quantitative mRNA expression analysis (Roche). Primers and probe information are summarized in **Supplementary Material 2**.

### 2.6 Gene knockdown

A lentiviral vector expressing shRNA against CRBN was prepared as previously described (14). In brief, a pRSI9-based control vector was prepared by removing the barcode and shRNA sequence from a pRSI9 plasmid isolated from a lentiviral shRNA library (Cellecta, Mountain View, CA, USA), and a synthetic double-stranded oligonucleotide against the following target sequence was cloned into a control vector: 5′-AAGTGCCAGATATTTCCTTCA-3′. Recombinant lentivirus was prepared using the resulting plasmid and the ViraPower Lentiviral Packaging Mix (Thermo Fisher, Waltham, MA, USA) and was transduced into cells for stable knockdown. Transduction was performed in the presence of 6 µg/mL polybrene. CRBN knockdown HuT102 cells were established by selection in the presence of 0.5 µg/mL puromycin. Lentivirus-expressing shRNAs targeting IKZF2 and SCR were also prepared according to the manufacturer’s instruction (Sigma-Aldrich, St. Louis, MO, USA). The IKZF2 interference sequences were as follows: shRNA-IKZF2-1(TRCN0000021929), 5′-CCCAGTTATAAGCTCAGCTTA-3′; shRNA-IKZF2-2(TRCN0000021930), 5′-CCAATGTGCTTATGGTACATA-3′; SCR, 5′-CAACAAGATGAAGAGCACCAA-3′.

### 2.7 DNA microarray gene expression analysis

Two ATL-related cell lines were treated with DMSO (control) or with 1.5 μmol/L LEN for 24 hours in triplicate. Total RNA was isolated using an RNeasy mini kit (Qiagen) and subjected to DNase treatment (RNase-Free DNase Set; Qiagen). A portion of the total RNA was reverse transcribed into cDNA. After the evaluation of RNA quality using an Agilent 2100 Bioanalyzer, each RNA was labeled using a Low Input Quick Amp Labeling Kit (one-color method; Agilent, Santa Clara, CA, USA) and hybridized using the SurePrint G3 Human GE microarray 8x60K version 2.0 (Agilent) for 17 hours at 65°C. Slides were scanned on the Agilent SureScan Microarray Scanner (G2600D) immediately after washing using a one-color scan setting for 8x60K array slides, and the scanned images were analyzed using the Feature Extraction Software 11.5.1.1 (Agilent).

### 2.8 Pathway enrichment analysis of the differentially expressed gene clusters

For the differentially expressed gene (DEG) pathway analysis, gene ontology (GO) functional enrichment analysis, which includes analysis of molecular function (MF), biological process (BP), and cellular component (CC) terms, and Kyoto Encyclopedia of Genes and Genomes (KEGG) pathway analysis of the differential expression genes were carried out using Metascape (http://metascape.org) (15), ggplot2, and VennDiagram package in R using the Xiantao website. A log2|(fold change)| >2 and p-value <0.05 were regarded as significant. These values were also confirmed by KEGG pathway enrichment analysis. Data from each array (cells treated with DMSO or LEN in triplicate) were median-normalized per chip. The data were then filtered based on the signal intensity and flagged values. Genes with |fold change| > 2 after LEN treatment of each cell line were identified using Student’s t-test with the Benjamini and Hochberg multiple testing correction to restrict the false discovery rate (FDR). Genes with corrected p-values less than 0.05 were considered to be differentially expressed. Commonly upregulated genes in HuT102 and OATL4 were subjected to KEGG pathway analysis using DAVID35 to investigate the key pathways affected by LEN. The KEGG terms with FDR < 0.05 were considered significant, and those with FDR < 0.1 were also listed for reference (16). The Gene Expression Omnibus (GEO) accession numbers for HuT102 and OATL4 are GSE221056 and GSE221062, respectively.

### 2.9 Adult T-cell leukemia/lymphoma xenograft mouse experiments

Female CB17 severe combined immunodeficiency (SCID) mice (body weight, 20 g; 20 per cell line) (CLEA Tokyo, Japan) were subcutaneously injected in the right flank with 5 × 10^6^ HuT102 cells in 50 μL saline containing 50 μL Matrigel (BD Biosciences, San Jose, CA, USA). When the tumor volume reached 100 mm^3^, mice were randomized into the control or lenalidomide group (n = 5 each), wherein each mouse was orally administered daily for 28 days following inoculation with 0.5% hydroxypropyl methylcellulose (Shin-Etsu Chemical, Tokyo, Japan) with 0.25% Tween 80 or lenalidomide (10, 50, and 100 mg/kg). Tumor diameters were measured using vernier calipers twice weekly. Data are expressed as the mean ± SEM.

### 2.10 Statistical calculation

The statistical significance between the control and LEN-treated groups (**Figure 6B**) or three drug treatments (**Figure 7**) was analyzed using one-way ANOVA followed by Dunnett’s test on GraphPad Prism 8.0. Results were considered significant when p < 0.05.

## 3 Results

### 3.1 LEN-mediated growth inhibition of ATL-related cell lines and biochemical characterization

**Figure 1:**
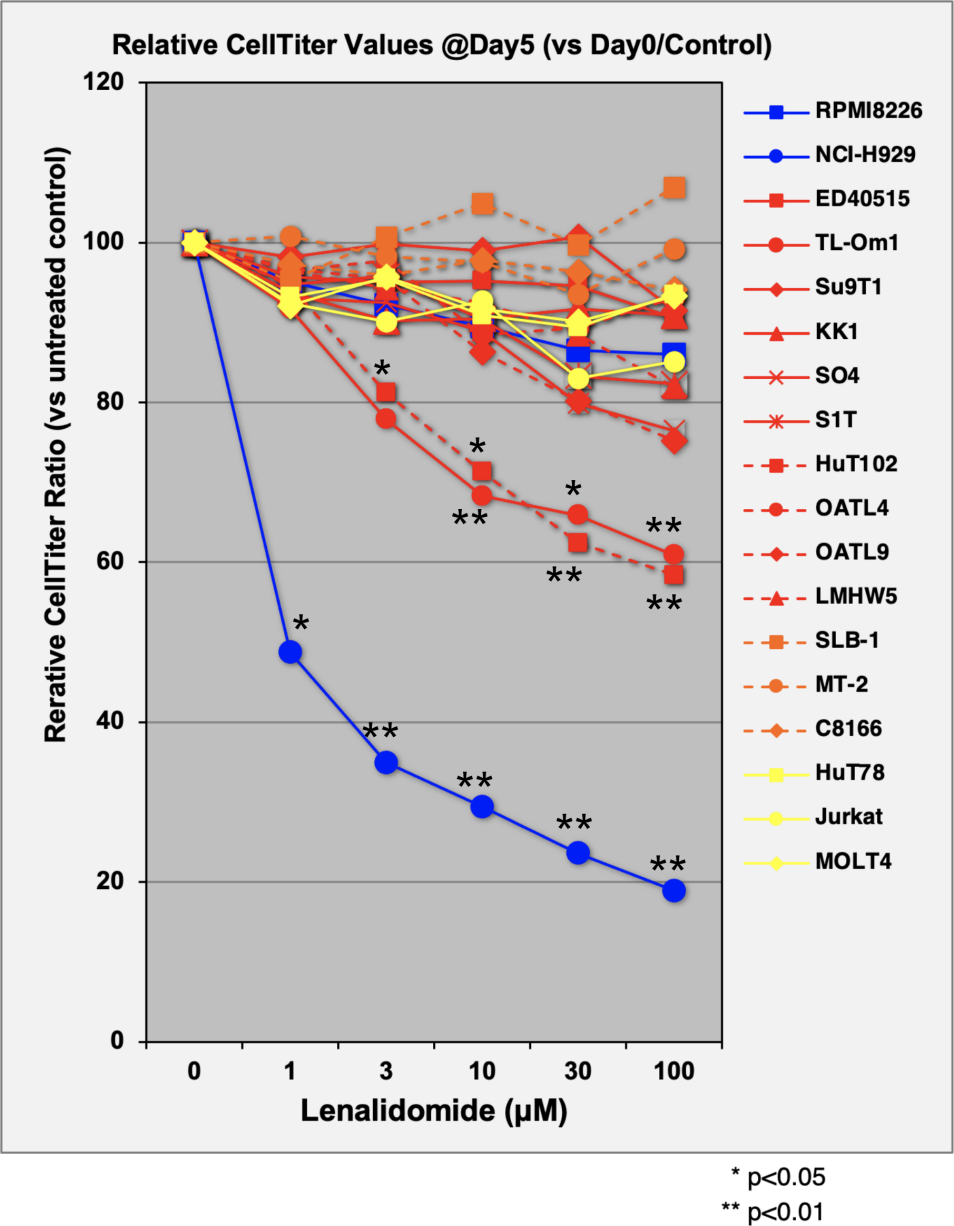
Evaluation of cellular proliferation inhibitory effects of LEN on ATL cell lines. Three control MM cells (blue lines), 10 ATL cells (red), three HTLV-1-transformed cells (orange), and three non-ATL cells (yellow) were cultured in media with or without lenalidomide (1 µM, 3 µM, 10 µM, 30 µM, and 100 µM) for 5 days. Proliferation activity of each cell line was evaluated using CellTiter Assay Kit (Promega). Average and standard deviation values were obtained from four independent experiments. p-Values were calculated by comparison of control versus treated. LEN, lenalidomide; ATL, adult T-cell leukemia/lymphoma; MM, multiple myeloma.

**Figure 2:**
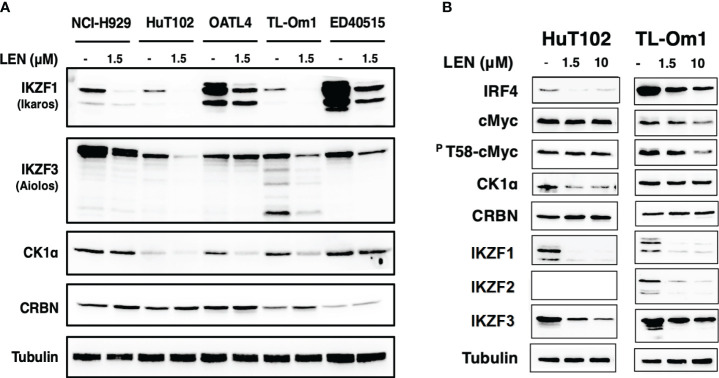
Enhanced LEN-induced downregulation of CRBN targets and their downstream effectors in the LEN-sensitive ATL cells. **(A)** Cellular lysates of LENsensitive (HuT102 and TL-Om1) and LEN-resistant (OATL4 and ED40515) with or without LEN treatment for 3 days were prepared and analyzed by Western blotting (WB) with specific monoclonal antibodies recognizing IKZF1/2/3, CK1a, and CRBN (Cell Signaling Technology, Danvers, MA, USA). LEN-sensitive NCI-H929 (MM) cell lysates were also placed as the positive control. Tubulin was probed with an anti-tubulin monoclonal antibody (Sigma) as the internal control. **(B)** HuT102 and TL-Om1 were treated with LEN (0 µM, 1.5 µM, and 10 µM) for 3 days, and cellular lysates were harvested for WB using monoclonal antibodies of IRF4, c-Myc, and PT58-c-Myc (Cell Signaling Technology) in addition to antibodies used in panel **(A)** LEN, lenalidomide; ATL, adult T-cell leukemia/lymphoma; MM, multiple myeloma.

The direct growth inhibitory effects of LEN were examined for 13 ATL-related cell lines and three non-ATL T-cell lines (**Figure 1**; **Supplementary Data 3A**) with MM-derived control cells (LEN-sensitive NCI-H929 and LEN-resistant RPMI-8226). HuT102 and TL-Om1 (showing statistically significant growth suppressive profiles (**Supplementary 3B, C**): LEN sensitive) and OATL4 and ED40515 (as LEN-resistant) were selected for further biochemical evaluation. The anti-tumor effects induced by LEN are mediated through the modification of the target specificity of CRBN, an E3 enzyme responsible for ubiquitinating IKZF1 and IKZF3 in MM cells (7, 8), as well as casein kinase 1 alpha (CK1α) in 5q-MDS (10). We, therefore, examined the LEN-CRBN-mediated target behavior among five ATL cell lines (**Figure 2A**). The basal expression of IKZF1 and IKZF3 seemed higher in the LEN-resistant OATL4 and ED40515 (**Figure 2A**, top two panels), and CRBN expression was lower in ED40515 than any other cell lines (**Figure 2A**, second from the bottom). While LEN-induced target degradation (IKZF1 and IKZF3) was clearly indicated in HuT102 and TL-Om1, it was much less efficient in resistant OATL4 and ED40515. IKZF1/3 control IRF4 expression and its downstream effector c-Myc are the main drivers for tumor cell growth in MM (7, 8). Effective IKZF1/3 degradation (**Figure 2B**, sixth and eighth panels) and downregulation of IRF4 (**Figure 2B**, top panel) were confirmed in LEN-sensitive HuT102 and TL-Om1. Additionally, abnormal expression of IKZF2 was also observed (**Figure 2B**, seventh from top) in HuT102 (IKZF2-deleted) and TL-Om1 (IKZF2 downregulated). Suppression of c-Myc and its phosphorylated forms was more significant in TL-Om1 (**Figure 2B**, second and third panels) than those of HuT102. CK1α suppression was oppositely significant in HuT102 (**Figure 2B**, fourth from the top). To note, CRBN expression was enhanced by LEN in both cell lines (middle panel).

### 3.2 CRBN is essential for LEN-induced growth inhibition, and IKZF2 contributes to LEN resistance in ATL-related cell lines

**Figure 3:**
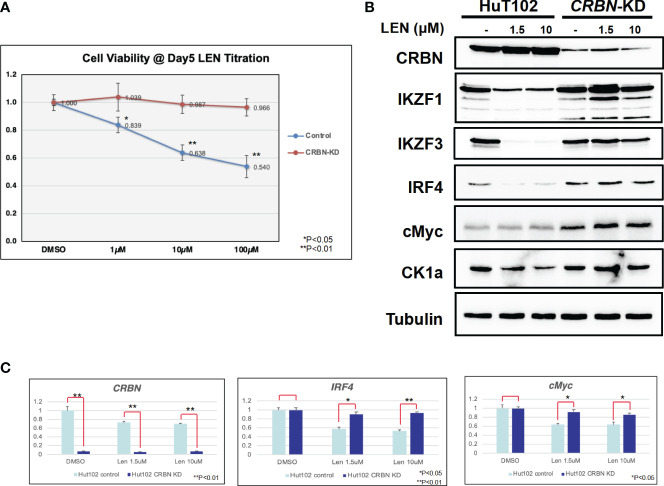
CRBN knockdown (KD) affects the LEN-induced anti-ATL activities in HuT102. **(A)** HuT102 (control: blue line) and its CRBN-knockdown variants (CRBN-KD: orange line) were treated with indicated concentrations of LEN (0 µM, 1 µM, 10 µM, and 100 µM) for 5 days, and their relative viability was compared using CellTiter values. **(B)** Western blotting of LEN-related targets in HuT102 (left) and CRBN-KD (right). Each cellular lysate was prepared after 3-day treatment of control DMSO (–) or 1.5 or 10 mM LEN. **(C)** Relative mRNA expression levels of CRBN, IRF4, and c-Myc in HuT102 and CRBN-KD. Total RNA was prepared after 3-day treatment of LEN at 0 µM, 1.5 µM, and 10 µM. LEN, lenalidomide; ATL, adult T-cell leukemia/lymphoma.

**Figure 4:**
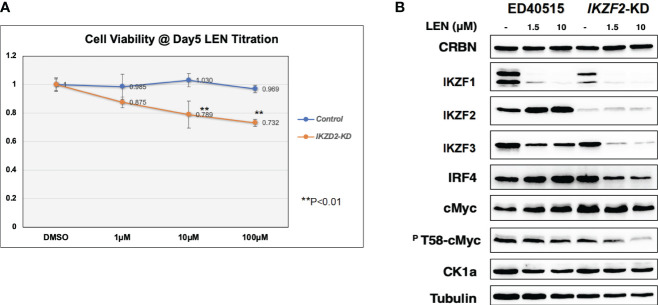
IKZF2-KD confers LEN sensitivity to LEN-resistant ED40515. **(A)** CellTiter analysis on control ED40515 (control: blue line) and IKZF2-knockdown variants (IKZF2-KD: orange line) with 5-day treatment of indicated concentrations of LEN (0 µM, 1 µM, 10 µM, and 100 µM). **(B)** WB analysis on CRBN targets in ED40515 and IKZF2-KD. Cell lysates were prepared after 3 days of treatment of LEN (0 µM, 1.5, and 10 µM) and probed with antibodies of CRBN, IKZF1/2/3, IRF4, c-Myc, pT58-c-Myc, and CK1α. Tubulin was probed as an internal control. KD, knockdown; LEN, lenalidomide; WB, Western blotting.

Since the anti-tumor effects of IMiDs are exerted through the E3 enzyme CRBN (7–10), lentivirus vector-based gene knockdown (KD) for CRBN was induced in LEN-sensitive HuT102 (Materials and Methods). While control HuT102 responded to 5 days of treatment of LEN (**Figure 3A**, blue line), the CRBN-KD variant became LEN-resistant (orange line). The expression of LEN-CRBN targets (IKZF1/3 and CK1α) was not diminished, downstream effectors (IRF4 and c-Myc) were enhanced in CRBN-KD variants (**Figure 3B**, right panels), and enhancements of both IRF4 and c-Myc mRNA levels were also confirmed (**Figure 3C**; light blue, HuT102; dark blue, CRBN-KD). When establishing LEN-resistant variants through culturing LEN-sensitive NCI-H929 cells in the LEN-containing medium, we observed a more than 10-fold increase in the expression of *IKZF2* gene (17). Considering the identified *IKZF2* deficiency in HuT102 (18), which exhibits LEN sensitivity, we subsequently examined the relevance of LEN resistance through *IKZF2* in ATL cells. *IKZF2*-KD variant was generated for LEN-resistant ED40515, and its LEN response was compared with that of control ED40515. The significant growth suppression (**Figure 4A**) was observed only in the *IKZF2*-KD variant, accompanied by downregulation of IKZF1/3, IRF4, and c-Myc (**Figure 4B**).

### 3.3 Distinct gene expression profiles between LEN-sensitive HuT102 and LEN-resistant OATL4 after LEN treatment

**Figure 5:**
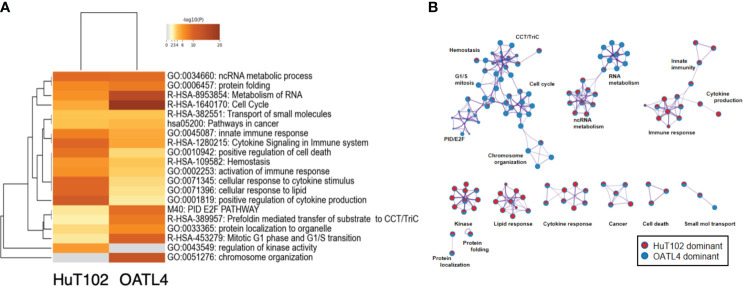
Distinct LEN-induced gene expression profiles between HuT102 and TL-Om1; 5 × 10^6^ of HuT102 cells or OATL4 cells were treated with 1.5 µM LEN for 24 hours, and total RNA was prepared for DNA microarray analysis. Differentially expressed gene (DEG) clusters are displayed with **(A)** the enrichment pathway heat map and **(B)** the pathway network of DEGs enriched in either HuT102 (red) or OATL4 (blue) (see ref. 15 for details). LEN, lenalidomide.

Reported targets of the LEN-CRBN axis in MM cells were similarly downregulated in LEN-responsive ATL cells (7, 8). Then, a DNA microarray was performed to assess the different effects of LEN inhibition in HuT102 and OATL4. The DEGs in LEN-sensitive HuT102 and LEN-resistant OATL4 were ranked, and functional annotation clustering was analyzed using the Metascape program (**Figure 5**; **Supplementary 5A, B, E, F**, **Supplementary 6**). The enriched ontology clusters (15) were displayed with the enrichment pathway heat map (**Figure 5A**). While the cytokine and lipid response pathways were significantly enriched in HuT102 cells, the cell cycle and metabolism of the RNA pathway were evident in OATL4 (**Figure 5A**; **Supplementary 5C, D**). Each enriched subset cluster was represented in the pathway network (**Figure 5B**; **Supplementary 5A**), along with a preponderance of either HuT102 or OATL4 (**Figure 5B**); statistical significance of these pathways was indicated in color gradations (**Supplementary 5B**). The enrichment analysis demonstrated that the pathway involved in immune response, cytokine production, kinase activation, and lipid responses was prevalent in HuT102 with 24-hour LEN treatment. The DEGs of OATL4, however, were enriched in cellular growth or gene expression such as Cell cycle, G1/S transition, chromosome organization, and molecular chaperone. The DAVID (KEGG pathway) analysis was also performed on differentially induced multiple genes in LEN-treated HuT102 and OATL4 (**Supplementary 4**–**12**) and reconfirmed quite different responses between the two cell lines (16). In contrast, HuT102 became more immune competent (TNF signaling pathway [FDA:1.74E-2], Cytosolic DNA-sensing pathway [FDA:3.49E-2], Chemokine signaling pathway [FDA:5.07E-2], and Toll-like receptor signaling pathway [FDA:5.55E-2]), which are opposite from the typical biological signatures of ATL-cells (3, 5, 6). OATL4 displayed more tumorigenic and anti-apoptotic responses to LEN treatment (Viral carcinogenesis [FDA:3.04E-11], Cell cycle FDA:1.48E-4], and Hepatitis B [FDA:2.43E-3]). Despite these differences, the Circos plot and the Venn diagram still showed overlapping DEGs (such as in the ncRNA processing pathway) between HuT102 and OATL4 under the effect of LEN (**Supplementary 6**). Additionally, an analysis of the response to LEN treatment was conducted in another LEN-resistant cell line, ED40515 (**Supplementary 14**). Remarkably, a substantial enrichment was observed in pathways associated with the negative regulation of DNA-binding transcription factor activity, responses to endoplasmic reticulum stress, and cellular responses to stress in ED40515 (**Supplementary 14A**). In contrast to HuT102, ED40515 displayed a clear emphasis on pathways related to inflammatory responses and the positive regulation of cell development (**Supplementary 14B**). Conversely, when compared to OATL4, the response pathways of ED40515 were notably centered around transcriptional misregulation in cancer and VEGFA-VEGFR2 signaling (**Supplementary 14C**). These findings suggest that during the later stages of ATL development, the ATL pathway becomes increasingly focused on cell growth following the disappearance of the Tax oncoprotein.

### 3.4 Orally administrated LEN blocked the growth of xenografted HuT102 cells in CB17-SCID mice

**Figure 6:**
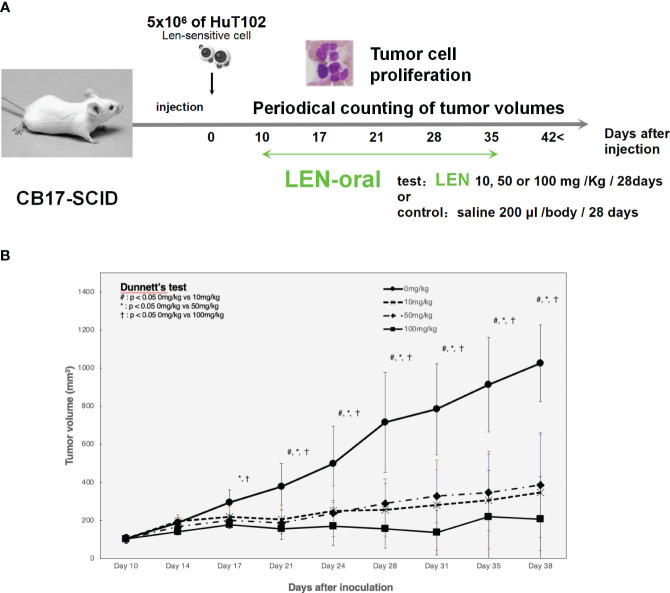
Oral administration of LEN suppresses tumorigenic growth of HuT102 cells xenografted in SCID mice. **(A)** The schematic representation of LENefficacy evaluation with HuT102 xenografted SCID mice experiment; 5 × 10^6^ HuT102 cells were injected subcutaneously into five SCID mice. After a formation of tumor lump (approximately 100 mm^3^ each), mice were orally administered LEN at 10 mg/kg, 50 mg/kg, and 100 mg/kg or saline for controls, 5 days a week for 8 weeks, and tumor sizes were measured weekly. **(B)** Average volume of tumor lumps in saline control (solid line with circle dots) and LEN-treated groups 10 mg/kg (altered dashed line with diamond dots), 50 mg/kg (even dashed line with X dots), and 100 mg/kg (solid line with square dots). Data are expressed as the mean ± SD of five mice. *p < 0.05, **p < 0.01 (analyzed using ANOVA followed by Dunnett’s test). BW, body weight; LEN, lenalidomide; SCID, severe combined immunodeficiency.

LEN efficacy was evaluated using an ATL cell-xenografted mouse model; 5 × 10^6^ of HuT-102 was inoculated in the right flank of CB17-SCID mice to let tumor cells grow to form tumor lumps (approximately 100 mm^3^), and three independent doses of LEN were orally administered once a day for 4 weeks (**Figure 6A**). The mean tumor volumes of HuT-102 cells in all three LEN-treated groups were significantly lower (even with the lowest dose of 10 mg/kg) than control throughout the experiment (**Figure 6B**), and no obvious adverse effects were observed (all individual mice treated with LEN showed no change in body weight, **Supplementary 15**).

### 3.5 Iberdomide, a new cereblon modulator, exerts its superior growth-suppressing effects on ATL cells

**Figure 7:**
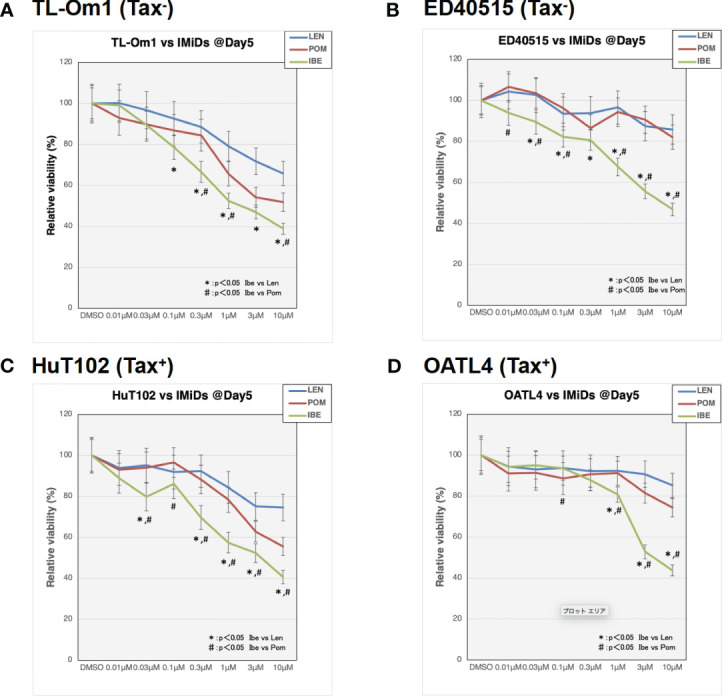
Distinct growth-suppressing effects of iberdomide (IBE) against ATL cells. ATL cell lines TL-Om1 **(A)**, ED40515 **(B)**, HuT102 **(C)**, and OATL4 **(D)** were treated with distinctive IMiDs: LEN (blue line), pomalidomide (POM; red), or CELMoD (IBE; green). Each cell line (5 × 10^5^ cells) was treated with three drugs at titrated concentrations (0.01 mM, 0.03 mM, 0.1 mM, 0.3 mM, 1 mM, 3 mM, and 10 mM) for 5 days. Data are expressed as the mean ± SD of triplicated experiments. p-Values were analyzed using ANOVA followed by Dunnett’s test. ATL, adult T-cell leukemia/lymphoma; IMiDs, immunomodulatory drugs; LEN, lenalidomide; CELMoD, cereblon modulator.

**Figure 8:**
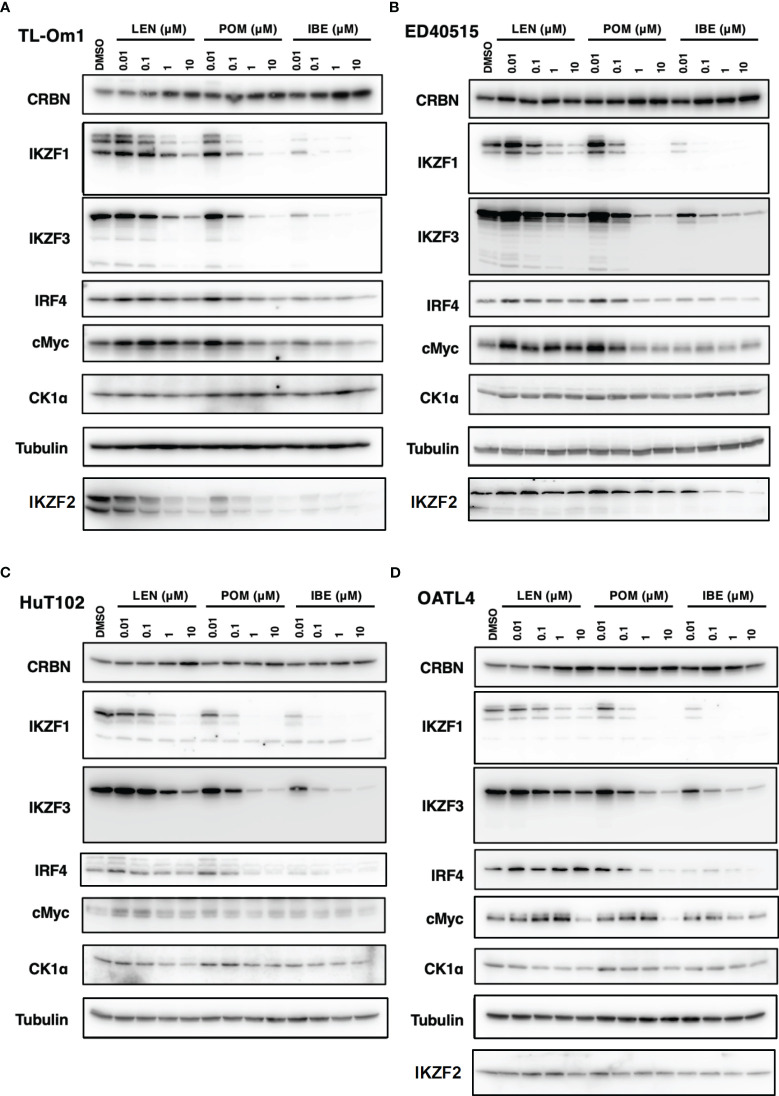
Distinct biochemical outcomes of CRBN targets after treatment of IMiDs or CELMoD in ATL cells. Cellular lysates of TL-Om1 **(A)** ED40515 **(B)**, HuT102 **(C)**, and OATL4 **(D)** treated with three drugs at titrated concentrations (0.01 µM, 0.1 µM, 1 µM, and 10 µM) were resolved and probed with antibodies of CRBN, IKZF1/3, IRF4, c-Myc, and CK1α. Tubulin was probed as an internal control. IMiDs, immunomodulatory drugs; CELMoD, cereblon modulator; ATL, adult T-cell leukemia/lymphoma.

As indicated in the clinical studies (11, 12), all our *in vitro* and *in vivo* experiments proved the “limited” efficacy of LEN against ATL-related cells. We, therefore, sought a better outcome against ATL cells with other IMiDs, POM and the CELMoD known as IBE (CC-220) (19). Among three drugs, only IBE reached its IC50 efficacy in all four ATL cell lines (**Figure 7A**, green lines). LEN (blue lines) and POM (orange lines) did not show significant differences from each other (**Supplementary 16**). The biochemical evaluation also proved superior effects of IBE against CRBN targets compared to the other two drugs (**Figure 8**; quantitation values are summarized in **Supplementary 17**). We also unexpectedly identified IBE-induced IKZF2 degradation even in LEN-resistant ED40515 (**Figure 8** and **Supplementary 17**). Although the growth suppression (**Figure 7**) and target degradation (**Figure 8**) were enhanced more by POM than those observed in LEN-treated HuT102 and TL-Om1, POM did not exert sufficient effects against LEN-resistant OATL4 and ED40515. In contrast, IBE suppressed all ATL cell growth below 50% (**Figure 7**) and degraded target proteins with 100- to 1,000-fold efficiency to LEN (**Figure 8** and **Supplementary 16**). IBE, to our surprise, downregulated IKZF2 efficiently for all ATL cells tested in this experiment (**Figure 8** and **Supplementary 16**). IMiDs or CELMoD did not affect the expression of viral oncogene HBZ (**Supplementary 18**). The IBE administration effect was indeed dependent on CRBN (**Supplementary 19**). In CRBN-KD cells, the expression of CRBN was reduced to approximately 20% (**Supplementary 19A, B**), resulting in significantly weakened growth suppression of IMiD-sensitive HuT102 (**Supplementary 19C**).

## 4 Discussion

Since random proviral integration of HTLV-1 and following extensive genetic (5) or epigenetic (20) alteration occurs in the host genome, pathogenetic forms of ATL are extensively diverse and complicated (21). Clinical manifestations of ATL are divided into at least four different forms, and its prognosis has been unsatisfactory (2). Although the first option of ATL treatments is still chemotherapy followed by hematopoietic stem cell transfusion (22), new treatments have been approved such as lenalidomide (11–13) and mogamulizumab (anti-CCR4 monoclonal antibody) (4) during the last decade.

**Figure 9:**
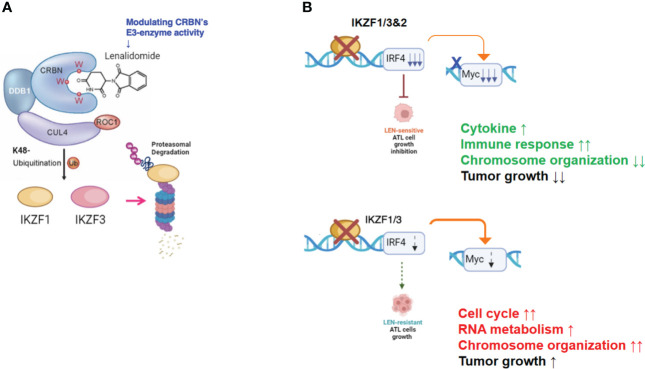
Diverse cellular responses in ATL cells to LEN treatment. **(A)** LEN modifies the target specificity of the E3 enzyme CRBN, leading to proteasomal degradation of newly targeted proteins such as IKZF1/3. **(B)** LEN-sensitive ATL cells effectively promote the degradation of IKZF1/3, leading to a substantial decrease in IRF4 and its downstream effector, Myc (upper panel). Consequently, these cells eventually halt their cellular growth. In contrast, LEN-resistant ATL cells retain IKZF1/3, possibly due to reduced CRBN activity, and continue to proliferate despite LEN treatment (lower panel). ATL, adult T-cell leukemia/lymphoma; LEN, lenalidomide.

In the current study, the biological and biochemical effects of three CRBN modulators against ATL-related cell lines were examined. The aim of the *in vitro* examination was to elucidate the efficacy mechanisms of LEN reported in the favorable results of LEN treatment on relapsed or recurrent ATL patients (11, 12). Only HuT102 and TL-Om1 among 13 tested ATL cell lines responded to LEN treatment moderately, but IC50 could not be obtained even with excessive doses (**Figure 1**). LEN-induced efficacy for several hematological malignancies was rationalized with modulation of the target specificity of E3 enzyme CRBN (7, 8). While LEN-responding HuT102 and TL-Om1 displayed quite similar biochemical outcomes such as IKZF1/3 degradation, LEN-resistant OATL4 and ED40515 maintained considerable amounts of those proteins (**Figure 2A**). IKZF1/3’s downstream effector IRF4 was also suppressed in LEN-responding cells (**Figure 2B**). In addition to these properties, minimized IKZF2 function was common in both HuT102 and TL-Om1. It is noteworthy that deletions or inversions in *IKZF2* gene were observed in 21% of ATL patients in Kataoka’s report (5). LEN-induced growth suppression mechanisms in ATL cells were confirmed by two knockdown experiments: HuT102-CRBN-KD (CRBN requirement for LEN-responsiveness, **Figure 3**) and ED40515-IKZF2-KD (complementary function of IKZF2 for IKZF1/3 and LEN-resistance, **Figure 4**). The *in vivo* efficacy of LEN was also confirmed with the HuT102 xenografted SCID mouse experiment (**Figure 6**). The anti-ATL effects mediated by IMIDs were distilled into a concise graphical summary (**Figure 9**).

According to these experimental data, the essential role of CRBN for LEN-induced efficacy and the potential role of IKZF2 for LEN resistance in ATL cells were implicated. Comparison of whole cell expression profiles between LEN-sensitive HuT102 and LEN-resistant OATL4 after LEN treatment provided additional supporting evidence of how LEN exerts its anti-tumor effects against ATL cells. Both cell lines displayed significantly altered mRNA expression profiles of as many as 2,192 (924 upregulated and 1,268 downregulated 2≧, HuT102) and 2,322 (917 upregulated and 1,404 downregulated 2≧, OATL4, **Supplementary 4**). The GO functional enrichment (**Figure 5**) and KEGG pathway analyses (**Supplementary 5A, B** and **Supplementary 7**–**12**) revealed extensively exclusive responses of both cells to LEN treatment. While HuT102 induced immune-activation signaling pathways (**Figures 5C, D**; **Supplementary 5**, **7**, **8**, **11**), which are basically absent in ATL cells (1–3, 5), and growth-suppressing properties (**Supplementary 8**), OATL4 induced rather anti-apoptotic and growth-promoting signals (**Figures 5A, B**; **Supplementary 5**, **9**–**11**). It is still elusive how LEN suppresses a certain population of ATL cells, but we conclude that the distinctive features in LEN-sensitive ATL cells could be attributed to the common CRBN-IKZF1/3-IRF4 downregulation axis and incompetency of complimentary function of IKZF2 for IKZF1/3 during LEN treatment (indicated as deletion/loss-of-function mutation, **Figure 2B**).

Despite these differences, the Circos plot and the Venn diagram still show the overlapping DEGs (such as in the ncRNA processing pathway) between HuT102 and OATL4 under the effect of LEN (**Supplementary 6**). Additionally, we conducted an analysis of the response to LEN treatment in another LEN-resistant cell line, ED40515 (**Supplementary 17**). Remarkably, we observed a substantial enrichment in pathways associated with the negative regulation of DNA-binding transcription factor activity, responses to endoplasmic reticulum stress, and cellular responses to stress in ED40515 (**Supplementary 17A**). In contrast to HuT102, ED40515 displayed a clear emphasis on pathways related to inflammatory responses and the positive regulation of cell development (**Supplementary 17B**). Conversely, when compared to OATL4, the response pathways of ED40515 were notably centered around transcriptional misregulation in cancer and VEGFA-VEGFR2 signaling (**Supplementary 17C**). These findings suggest that during the later stages of ATL development, the ATL pathway becomes increasingly focused on cell growth following the disappearance of the Tax oncoprotein.

It is still elusive how LEN suppresses a certain population of ATL cells, but we conclude that the distinctive features in LEN-sensitive ATL cells could be attributed to the common CRBN-IKZF1/3-IRF4 downregulation axis and incompetency of complimentary function of IKZF2 for IKZF1/3 during LEN treatment (indicated as deletion/loss-of-function mutation, **Figure 2B**). Likewise, refractory/relapsed MM patients (23) and the majority of ATL patients responded poorly to LEN treatment (11–13). One way to overcome this drug resistance is additional input such as monoclonal antibodies (4), which seems effective. Most ATL cells express a chemokine receptor CCR4, an intrinsic marker of regulatory T cells, on their cellular surfaces through the HBZ-FoxP3 pathway. A humanized monoclonal anti-CCR4 antibody mogamulizumab is a novel therapeutic agent for ATL (4). Even though classic and novel therapies are applicable to ATL, its multifaceted pathogenic features urgently require additional therapeutic options. A phase I/II study combining mogamulizumab and lenalidomide for CCR4-positive relapsed/refractory aggressive ATL is undergoing (24). It is also worth examining the combinatory use of other newly developed anti-cancer drugs such as Hsp90 inhibitors (25, 26) or epigenetic inhibitors (27, 28). In this report, we further assessed new-generation IMiDs, POM (8) and CELMoD IBE (CC-220) (29), which have superior IKZF1/3 degradation activity. The plasma Cmax values of LEN, POM, and IBE used in clinical practice are 1.9–3.1 µM (13), 180–220 nM, and (30) 3.3–6.6 nM (31), respectively. In our experimental concentration range, the proliferation inhibitory activity was hardly observed in the *in vitro* assays. However, the target molecules are significantly degraded, and a tendency to suppress the expression of IRF4 and cMyc, which drive the proliferation of ATL cells, was observed. Experiments using mice show effects, but it takes 2 weeks for them to be observed. Therefore, considering these observations collectively, it is possible to expect the pharmacological effects of IMiDs (i.e., proliferation inhibitory activity). However, a common feature among patients who responded to LEN treatment is the clear observation of hematologic and immunologic responses, including both efficacy and intake effects (11–13, 32). Understanding the host’s drug responsiveness in more detail remains a challenge that requires further exploration. While the primary weakness of this research lies in the absence of growth inhibition within the clinical dosage range of LEN, there were notable observations of the induced biochemical response within cells. Specifically, the induction of degradation of CRBN’s targets and the differential whole gene expression profiles between LEN-sensitive and LEN-resistant cell lines were observed at almost equal clinical doses of LEN. Furthermore, the observed growth inhibition of tumor cells in murine *in vivo* experiments, where no immune response can be expected, may to some extent mirror our biochemical assessments.

In summary, the molecular mechanisms through which IMiDs suppress the growth of ATL cells can be outlined as follows: LEN effectively reduces the expression of its target proteins in ATL cells by altering the target specificity of CRBN, leading to the degradation of IKZF1/3 (as shown in **Figure 9A**). However, the effectiveness of suppressing downstream effectors like IRF4 and c-Myc varies significantly between LEN-sensitive cells, which exhibit re-activation of immune responses, and LEN-resistant cells, which display enhanced cell growth signals related to processes such as the cell cycle, RNA metabolism, and chromosome organization (as depicted in **Figure 9B**). This deficiency in suppression can potentially be overcome with the use of IBE, as demonstrated in **Figures 7** and **8**.

While LEN has demonstrated its effectiveness as a maintenance treatment for aggressive ATL patients who have undergone chemotherapy (13), there is a growing concern regarding the risk of second primary malignancies, a phenomenon reported in patients with plasma cell myeloma or myelodysplastic syndrome receiving long-term IMiD treatment (33). Our experimental data support the hypothesis that LEN resistance arises due to its limited impact on IKZF2 degradation, offering an explanation for the increased risk of second primary malignancies in ATL patients undergoing prolonged LEN treatment. Therefore, it is highly preferable to find a more efficient CRBN modulator to shorten the treatment duration for ATL patients.

Our research demonstrates that the novel IMiDs/CELMoD combination, IBE, holds promise as a potential therapeutic agent for ATL, offering broader and more profound growth suppression in ATL cells and efficient degradation of IKZF2, a result not observed with LEN or POM treatments. This study underscores the novel and highly effective application of IBE in the context of LEN-resistant, recurrent/refractory ATL (34).

## Data availability statement

The datasets presented in this study can be found in online repositories. The names of the repository/repositories and accession number(s) can be found in the article/**Supplementary Material**.

## Ethics statement

This study was carried out in strict accordance with the recommendations in the Guidelines for Proper Conduct of Animal Experiments, Science Council of Japan (http://www.scj.go.jp/en/animal/index.html). All procedures involving animals and their care were approved by the Animal Care Committee of Miyazaki University in accordance with the Regulations for Animal Experiments in Miyazaki University (approval ID: 2011-505-8).

## Author contributions

YW: Data curation, Investigation, Methodology, Software, Validation, Writing – original draft. SS: Data curation, Investigation, Methodology, Validation, Writing – original draft. HI: Conceptualization, Data curation, Funding acquisition, Investigation, Methodology, Project administration, Resources, Supervision, Validation, Writing – original draft, Writing – review & editing. JY: Investigation, Methodology, Validation, Writing – review & editing. EI: Investigation, Methodology, Validation, Writing – original draft. NF: Investigation, Methodology, Data curation, Writing – original draft. TI: Investigation, Methodology, Writing – original draft. MH: Conceptualization, Funding acquisition, Resources, Writing – review & editing. MO: Resources, Writing – review & editing. YT: Data curation, Methodology, Validation, Writing – review & editing. NH: Data curation, Validation, Writing – review & editing. MM: Validation, Writing – review & editing. SH: Resources, Writing – review & editing. SK: Methodology, Writing – review & editing. MS: Methodology, Resources, Writing – review & editing. KA: Supervision, Validation, Writing – review & editing. AN: Funding acquisition, Resources, Writing – review & editing. HH: Conceptualization, Resources, Writing – review & editing. KM: Conceptualization, Formal analysis, Funding acquisition, Methodology, Validation, Writing – review & editing, Writing – original draft.
