# Supplementary material for: IMiD/CELMoD-induced growth suppression of adult T-cell leukemia/lymphoma cells via cereblon through downregulation of target proteins and their downstream effectors
Source: Front Oncol. 2024 Jan 24;13:1272528. doi: 10.3389/fonc.2023.1272528 (PMC10853999; doi:10.3389/fonc.2023.1272528)
Supplement: Supplementary file 8 [file Presentation_1.pptx]

## Slide 1
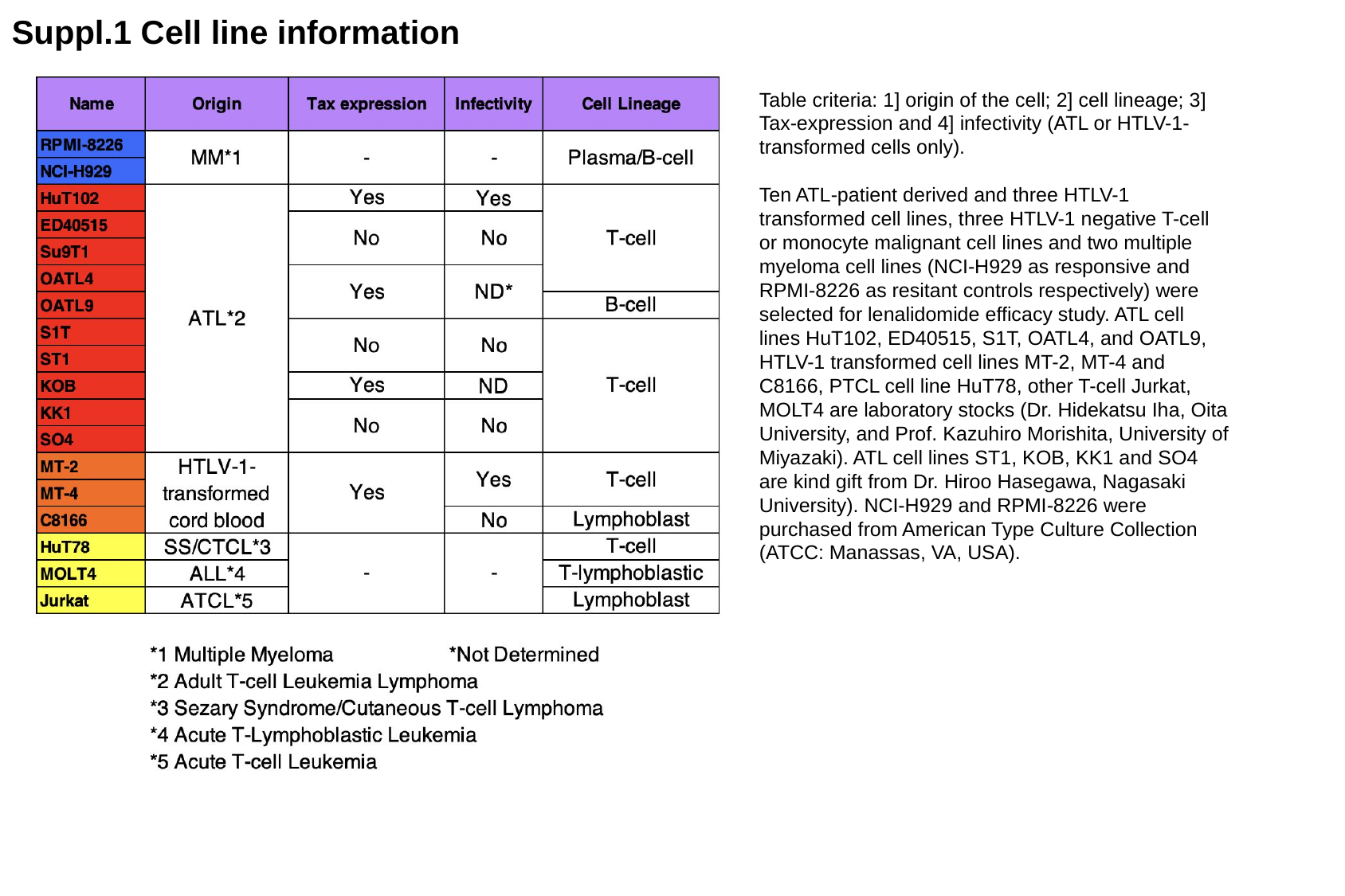

Suppl.1 Cell line information
Table criteria: 1] origin of the cell; 2] cell lineage; 3] Tax-expression and 4] infectivity (ATL or HTLV-1-transformed cells only).
Ten ATL-patient derived and three HTLV-1 transformed cell lines, three HTLV-1 negative T-cell or monocyte malignant cell lines and two multiple myeloma cell lines (NCI-H929 as responsive and RPMI-8226 as resitant controls respectively) were selected for lenalidomide efficacy study. ATL cell lines HuT102, ED40515, S1T, OATL4, and OATL9, HTLV-1 transformed cell lines MT-2, MT-4 and C8166, PTCL cell line HuT78, other T-cell Jurkat, MOLT4 are laboratory stocks (Dr. Hidekatsu Iha, Oita University, and Prof. Kazuhiro Morishita, University of Miyazaki). ATL cell lines ST1, KOB, KK1 and SO4 are kind gift from Dr. Hiroo Hasegawa, Nagasaki University). NCI-H929 and RPMI-8226 were purchased from American Type Culture Collection (ATCC: Manassas, VA, USA).

## Slide 2
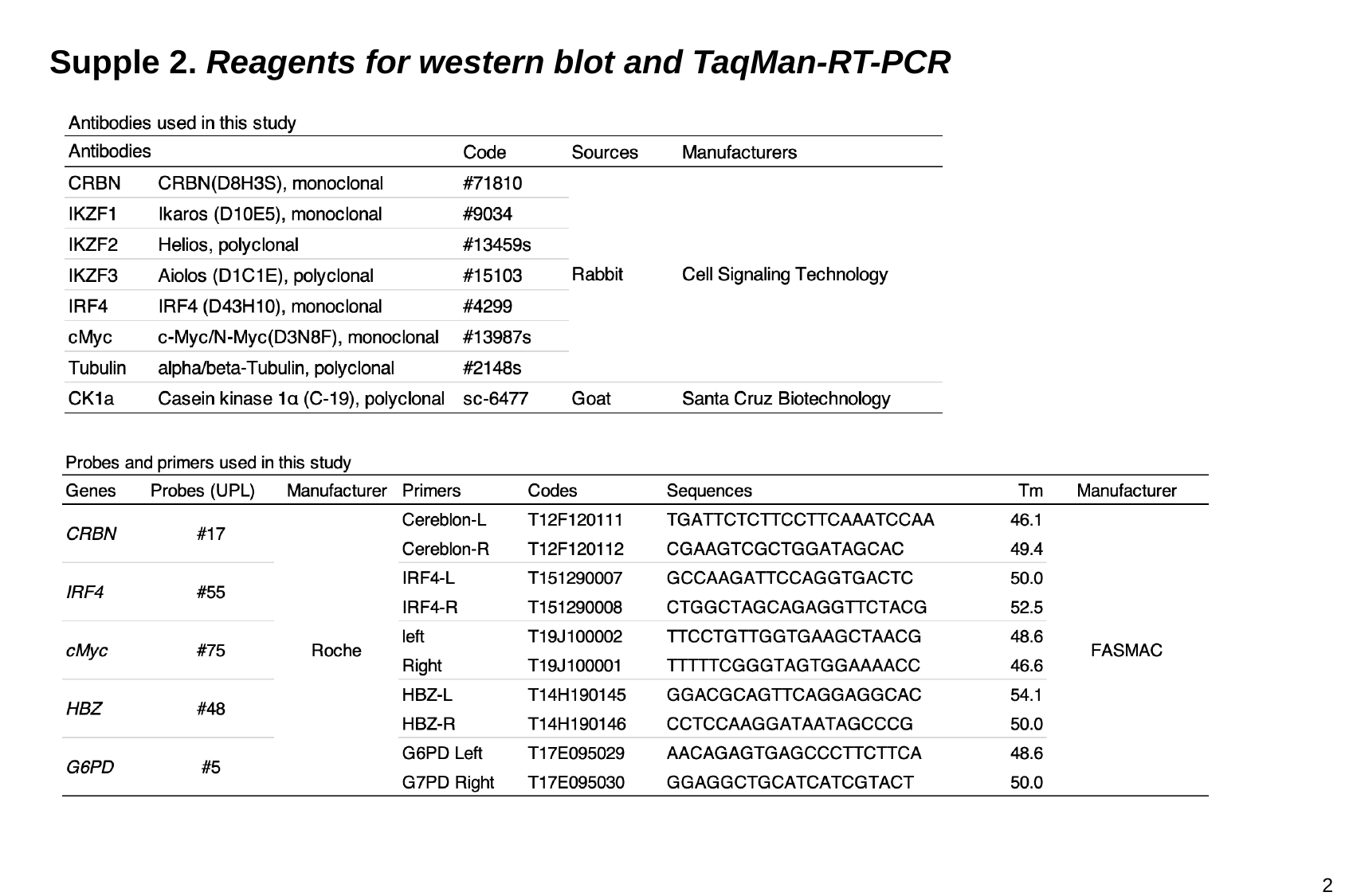

Supple 2. Reagents for western blot and TaqMan-RT-PCR
2

## Slide 3
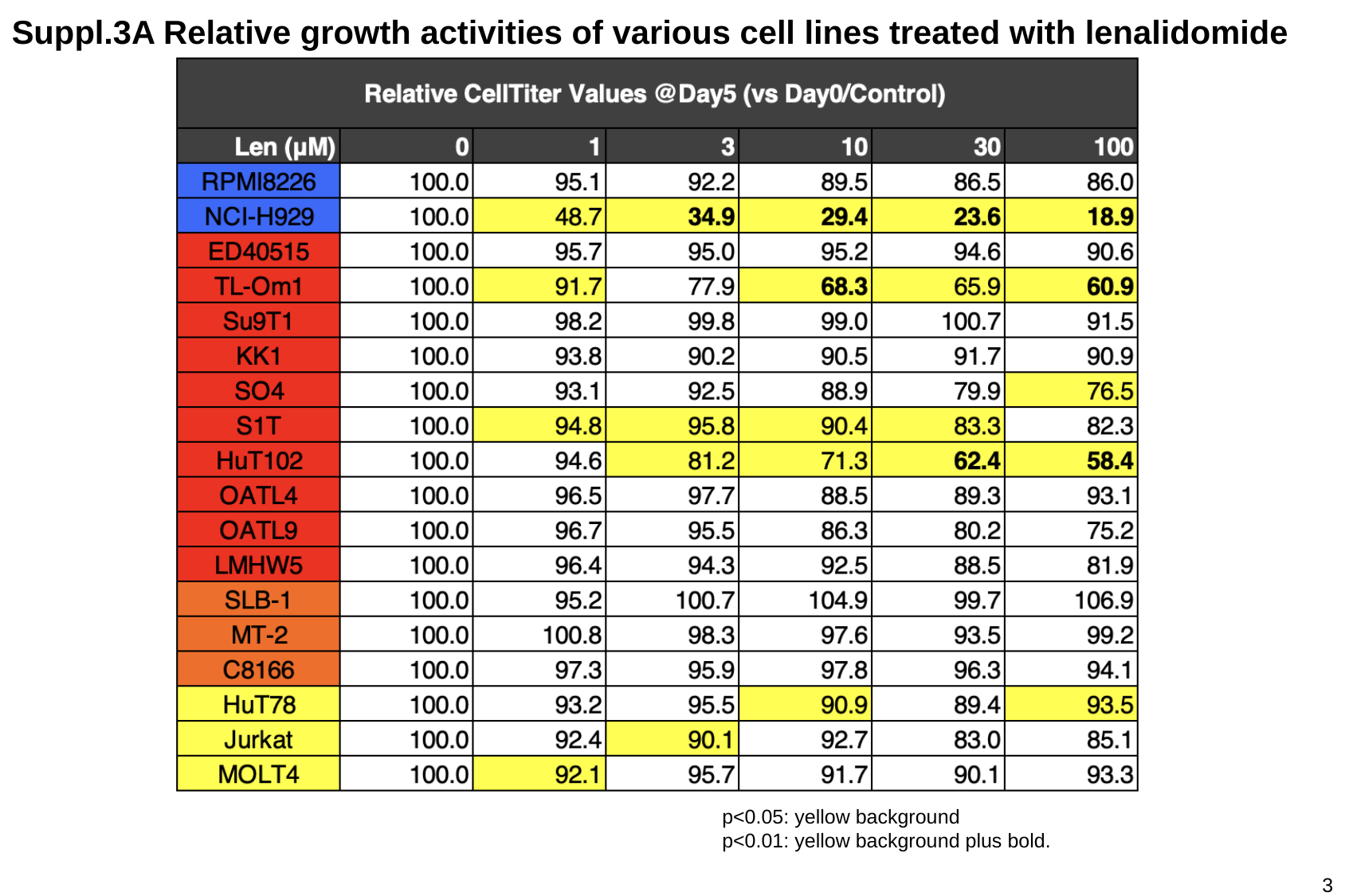

Suppl.3A Relative growth activities of various cell lines treated with lenalidomide
p<0.05: yellow background
p<0.01: yellow background plus bold.
3

## Slide 4
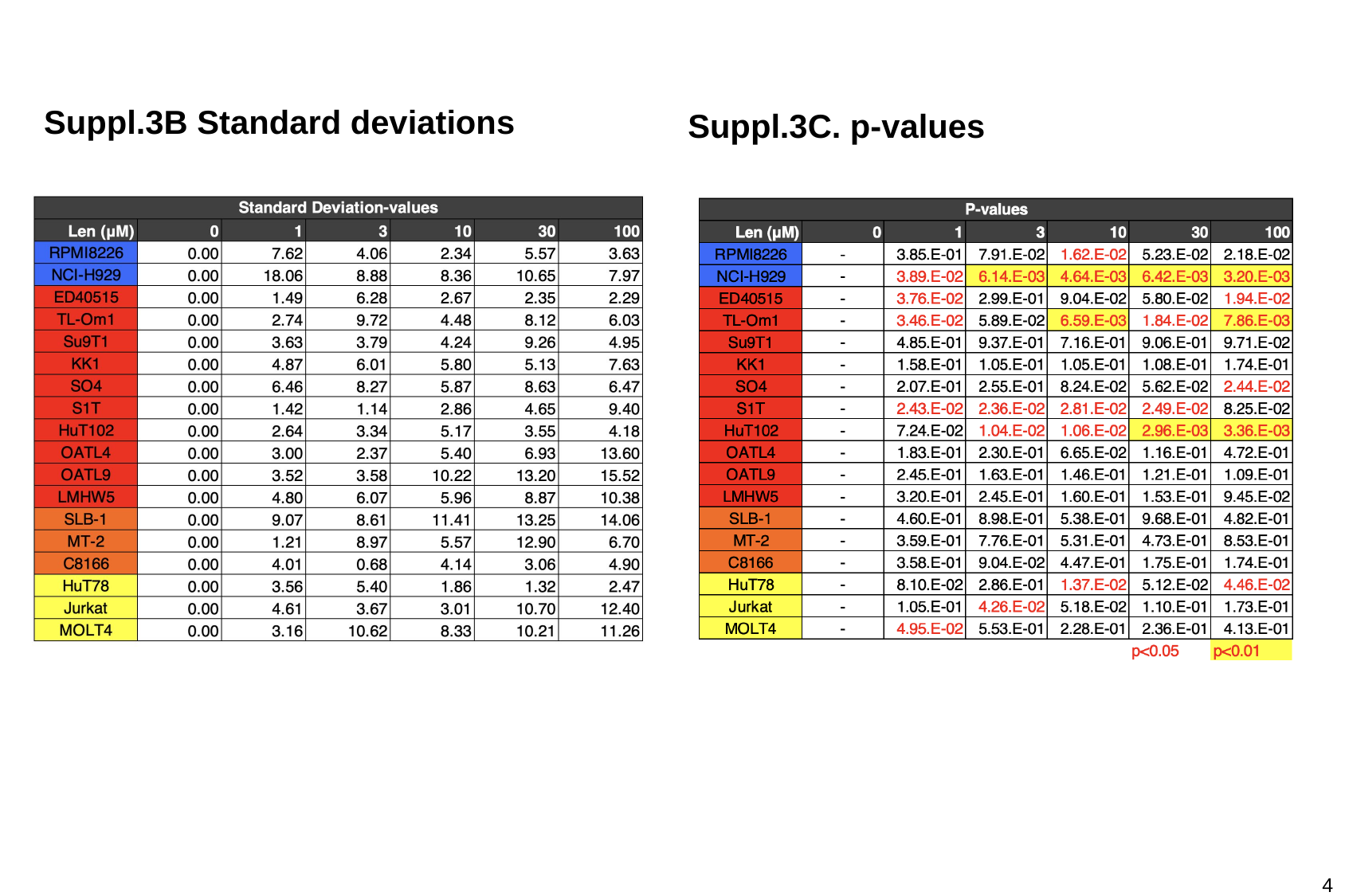

Suppl.3B Standard deviations
Suppl.3C. p-values
4

## Slide 5
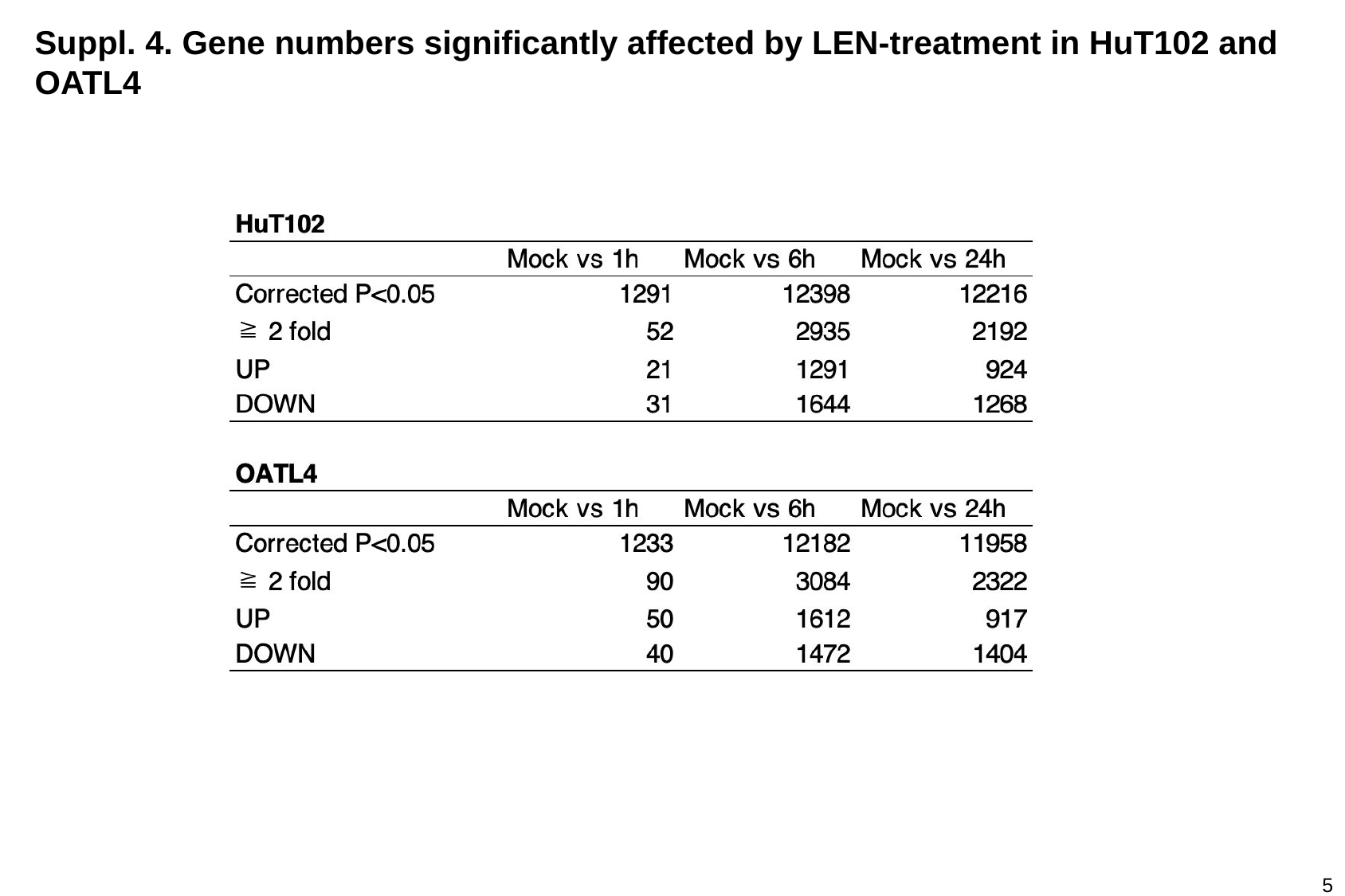

Suppl. 4. Gene numbers significantly affected by LEN-treatment in HuT102 and OATL4
5

## Slide 6
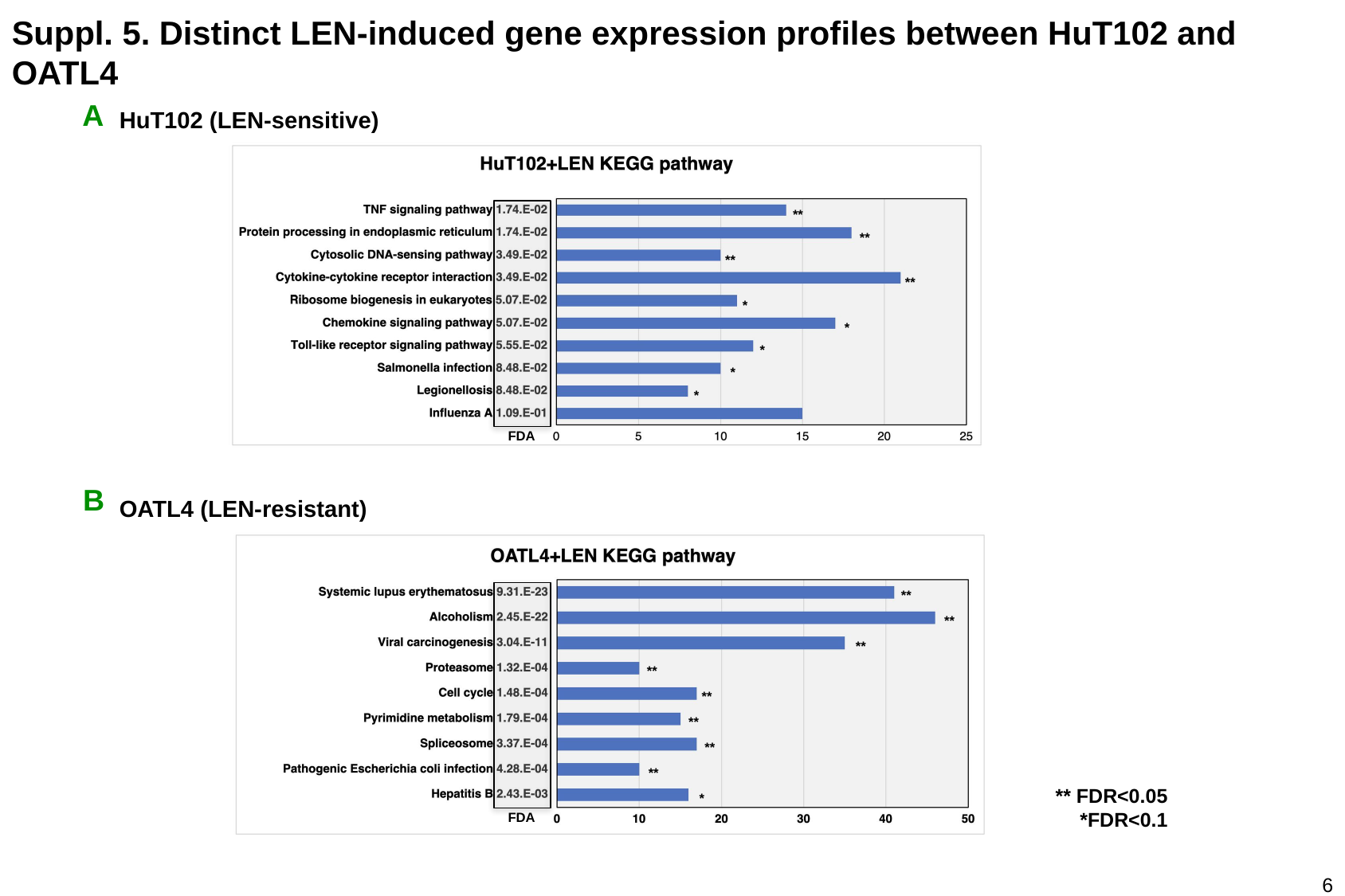

Suppl. 5. Distinct LEN-induced gene expression profiles between HuT102 and OATL4
A
HuT102 (LEN-sensitive)
**
**
**
**
*
*
*
*
*
FDA
B
OATL4 (LEN-resistant)
**
**
**
**
**
**
**
**
** FDR<0.05
 *FDR<0.1
*
FDA
6

## Slide 7
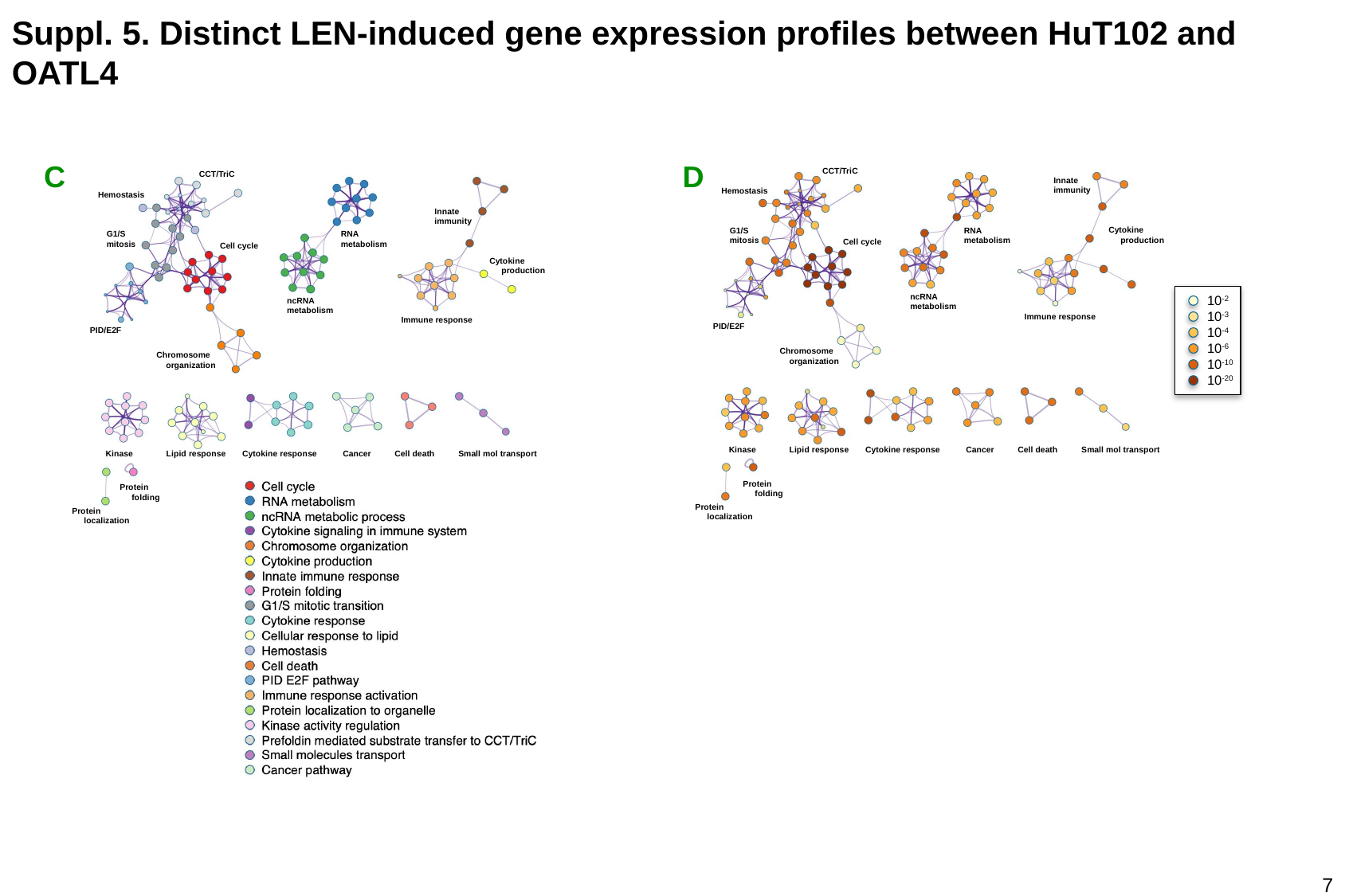

Suppl. 5. Distinct LEN-induced gene expression profiles between HuT102 and OATL4
C
D
CCT/TriC
CCT/TriC
Innate
immunity
 Cytokine
 production
Hemostasis
Hemostasis
Innate
immunity
 Cytokine
 production
G1/S
mitosis
RNA
metabolism
G1/S
mitosis
RNA
metabolism
Cell cycle
Cell cycle
ncRNA
metabolism
10-2
10-3
10-4
10-6
10-10
10-20
ncRNA
metabolism
Immune response
Immune response
PID/E2F
PID/E2F
Chromosome
 organization
Chromosome
 organization
Kinase Lipid response Cytokine response Cancer Cell death Small mol transport
Kinase Lipid response Cytokine response Cancer Cell death Small mol transport
 Protein
 folding
Protein
 localization
 Protein
 folding
Protein
 localization
7

## Slide 8
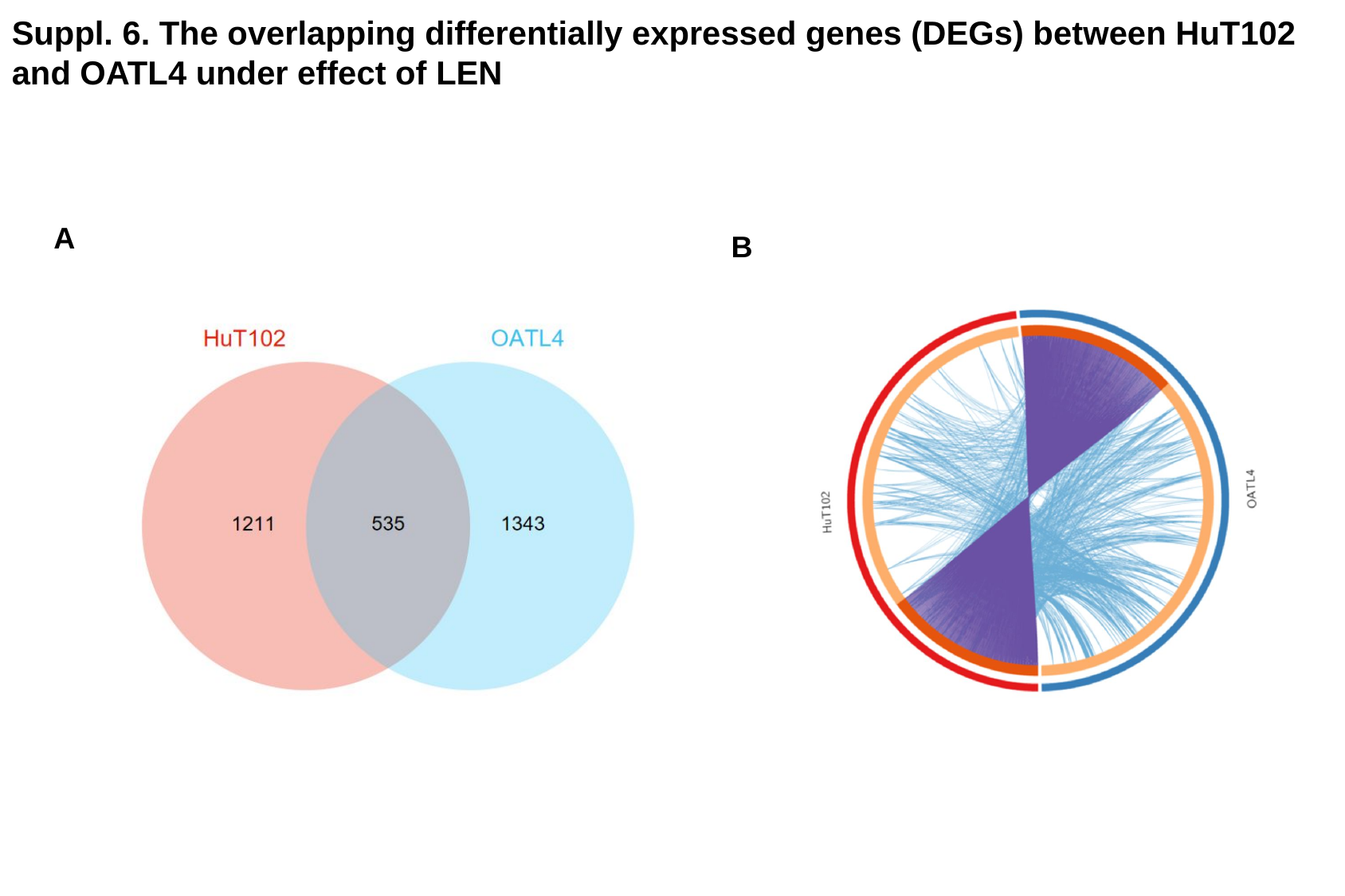

Suppl. 6. The overlapping differentially expressed genes (DEGs) between HuT102 and OATL4 under effect of LEN
A
B

## Slide 9
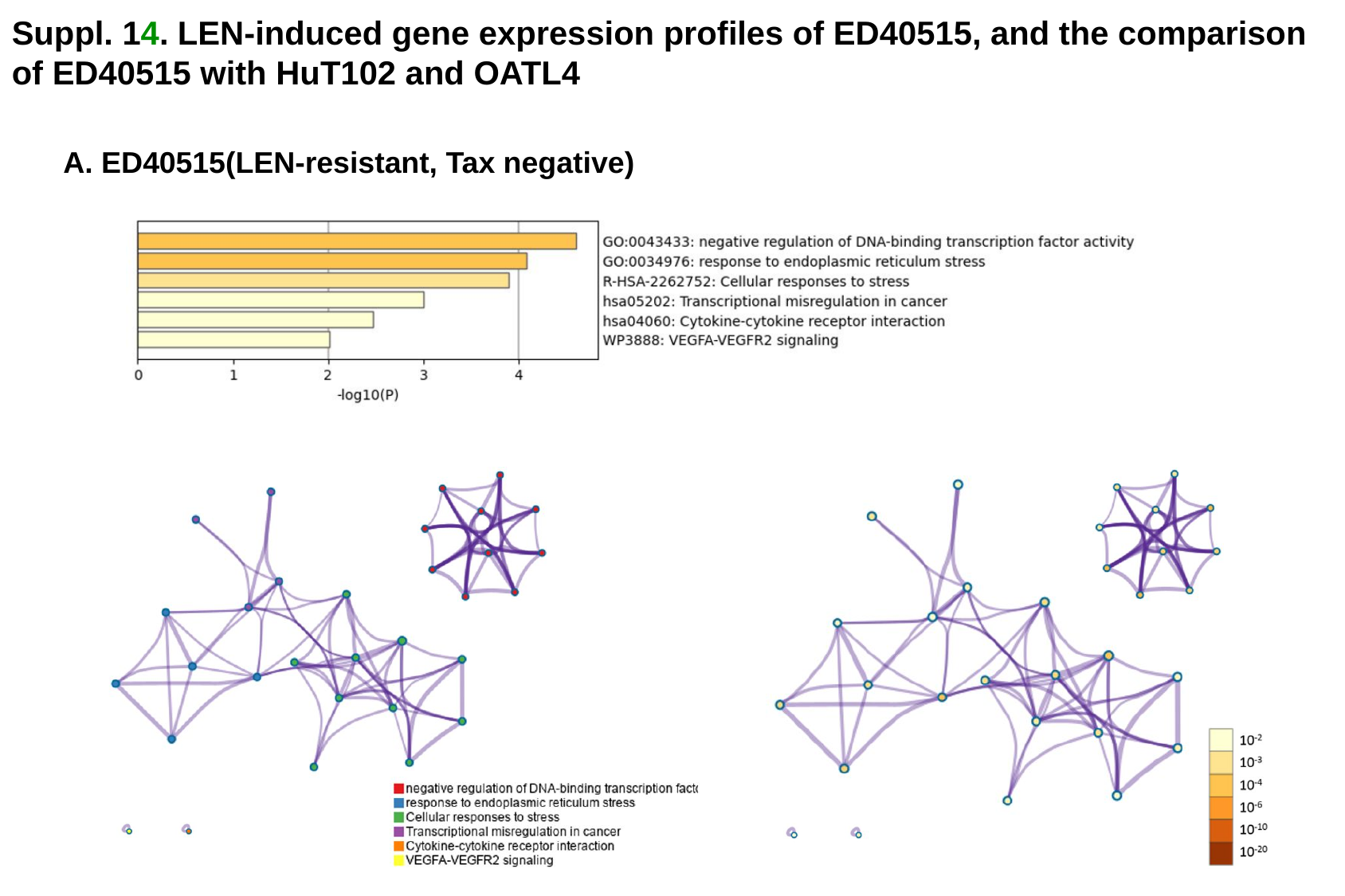

Suppl. 14. LEN-induced gene expression profiles of ED40515, and the comparison of ED40515 with HuT102 and OATL4
A. ED40515(LEN-resistant, Tax negative)

## Slide 10
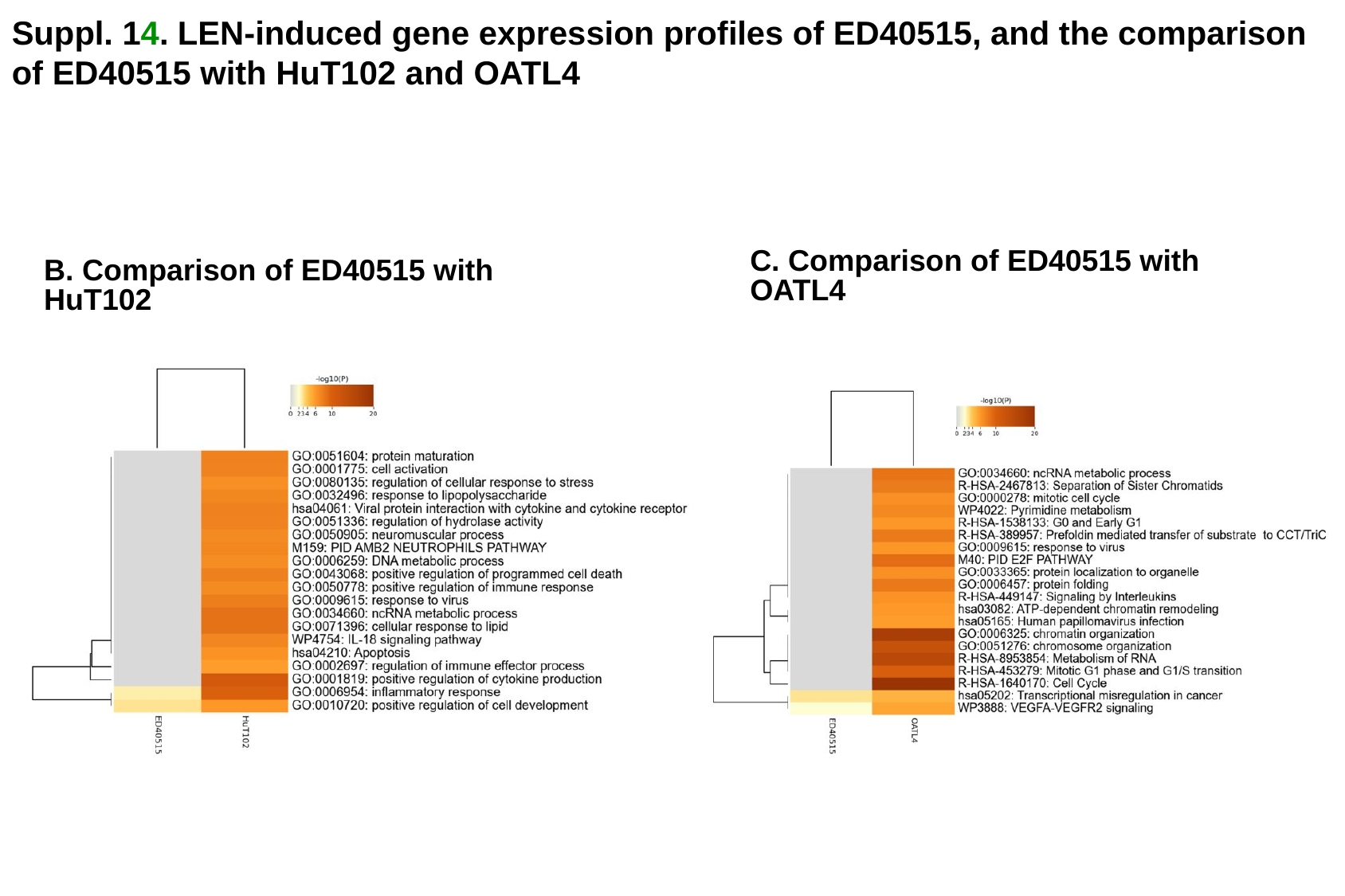

Suppl. 14. LEN-induced gene expression profiles of ED40515, and the comparison of ED40515 with HuT102 and OATL4
C. Comparison of ED40515 with OATL4
B. Comparison of ED40515 with HuT102

## Slide 11
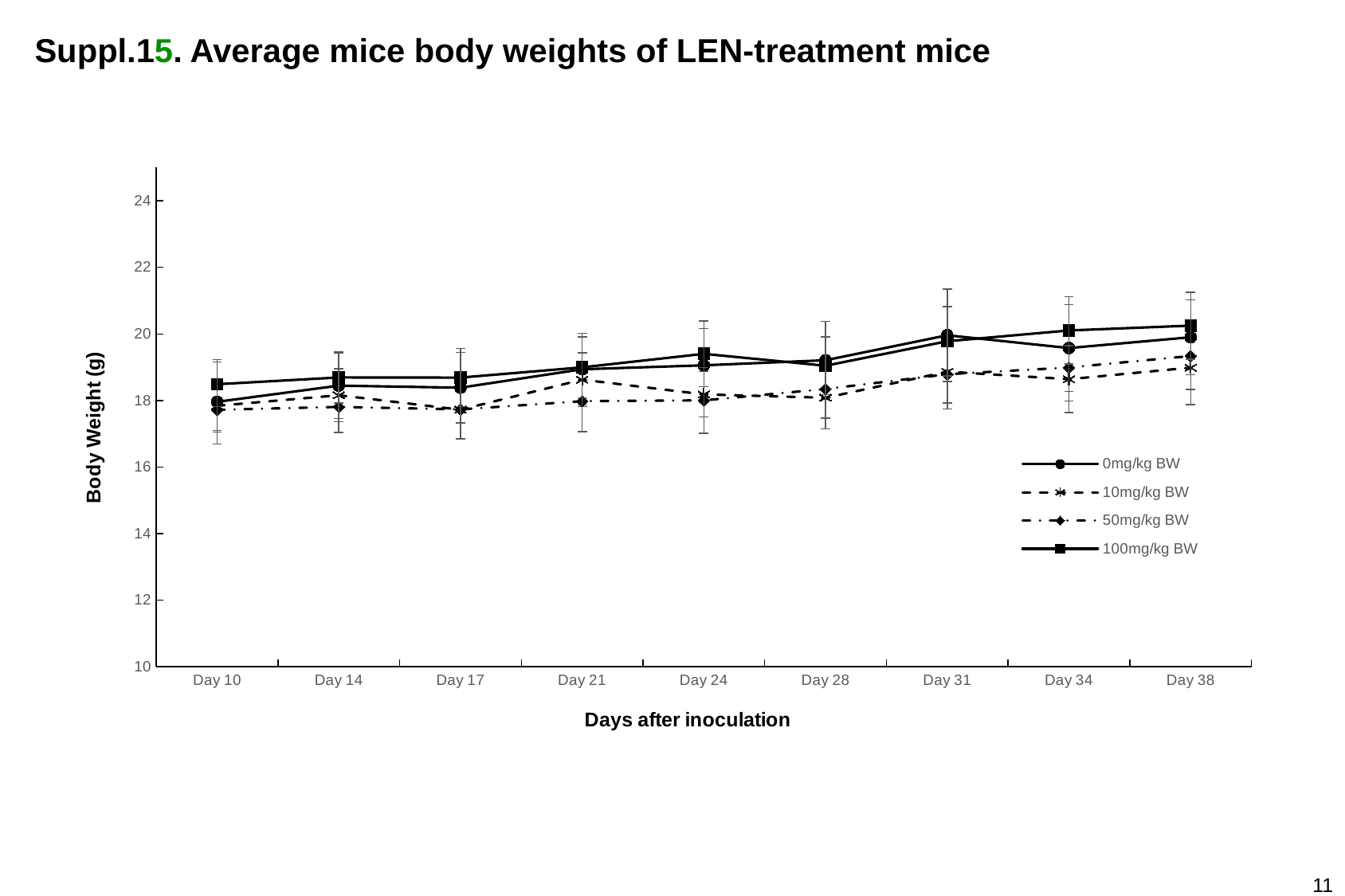

Suppl.15. Average mice body weights of LEN-treatment mice
### Chart
| Category | 0mg/kg BW | 10mg/kg BW | 50mg/kg BW | 100mg/kg BW |
|---|---|---|---|---|
| Day 10 | 17.96 | 17.84 | 17.72 | 18.488 |
| Day 14 | 18.446 | 18.158 | 17.808 | 18.692 |
| Day 17 | 18.384 | 17.722 | 17.732 | 18.688 |
| Day 21 | 18.938 | 18.626 | 17.98 | 18.996 |
| Day 24 | 19.056 | 18.184 | 18.004 | 19.402 |
| Day 28 | 19.21 | 18.082 | 18.344 | 19.044 |
| Day 31 | 19.96 | 18.866 | 18.788 | 19.78 |
| Day 34 | 19.576 | 18.64 | 18.992 | 20.104 |
| Day 38 | 19.9 | 18.988 | 19.336 | 20.25 |11

## Slide 12
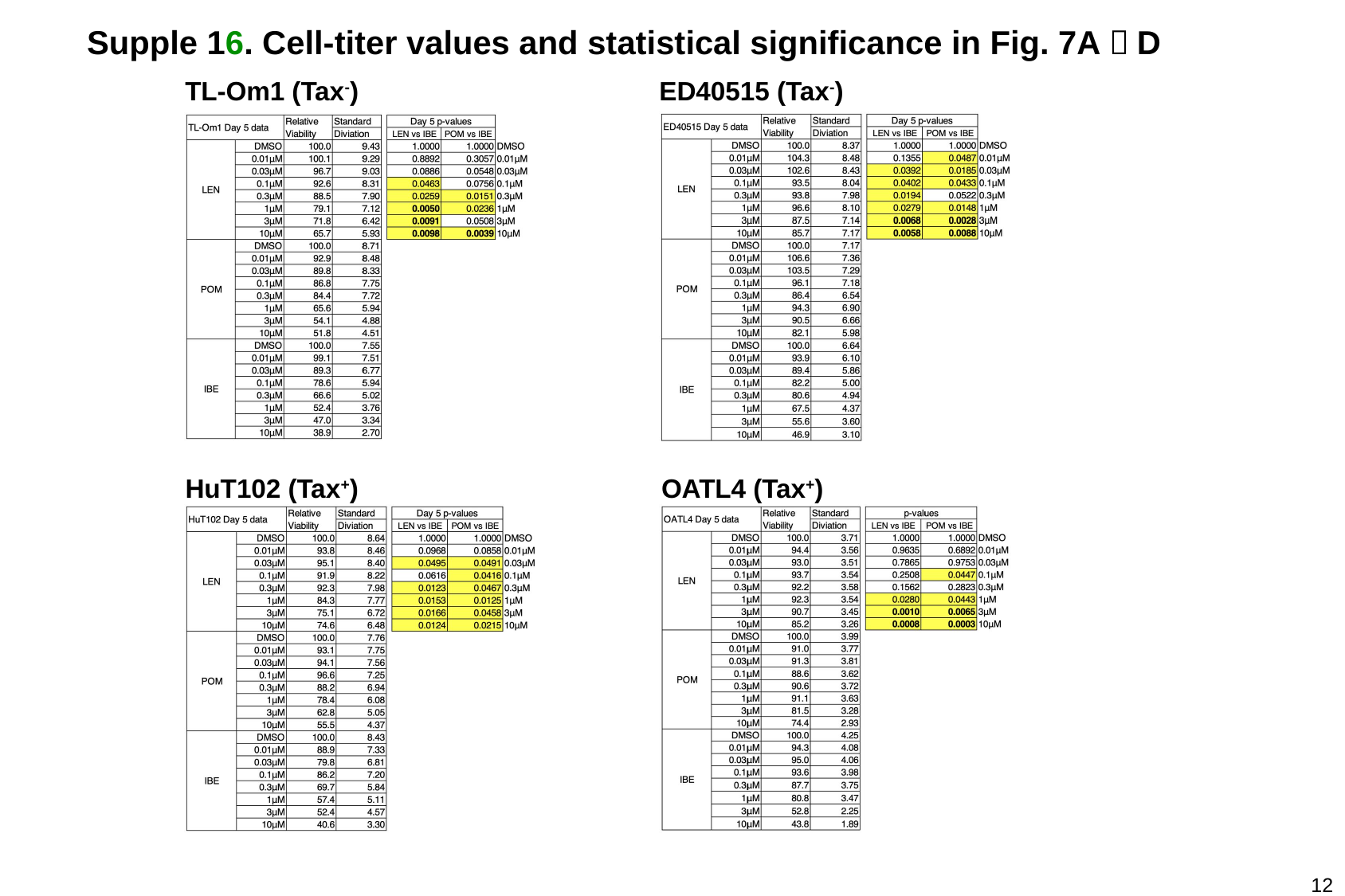

Supple 16. Cell-titer values and statistical significance in Fig. 7A〜D
TL-Om1 (Tax-)
ED40515 (Tax-)
HuT102 (Tax+)
OATL4 (Tax+)
12

## Slide 13
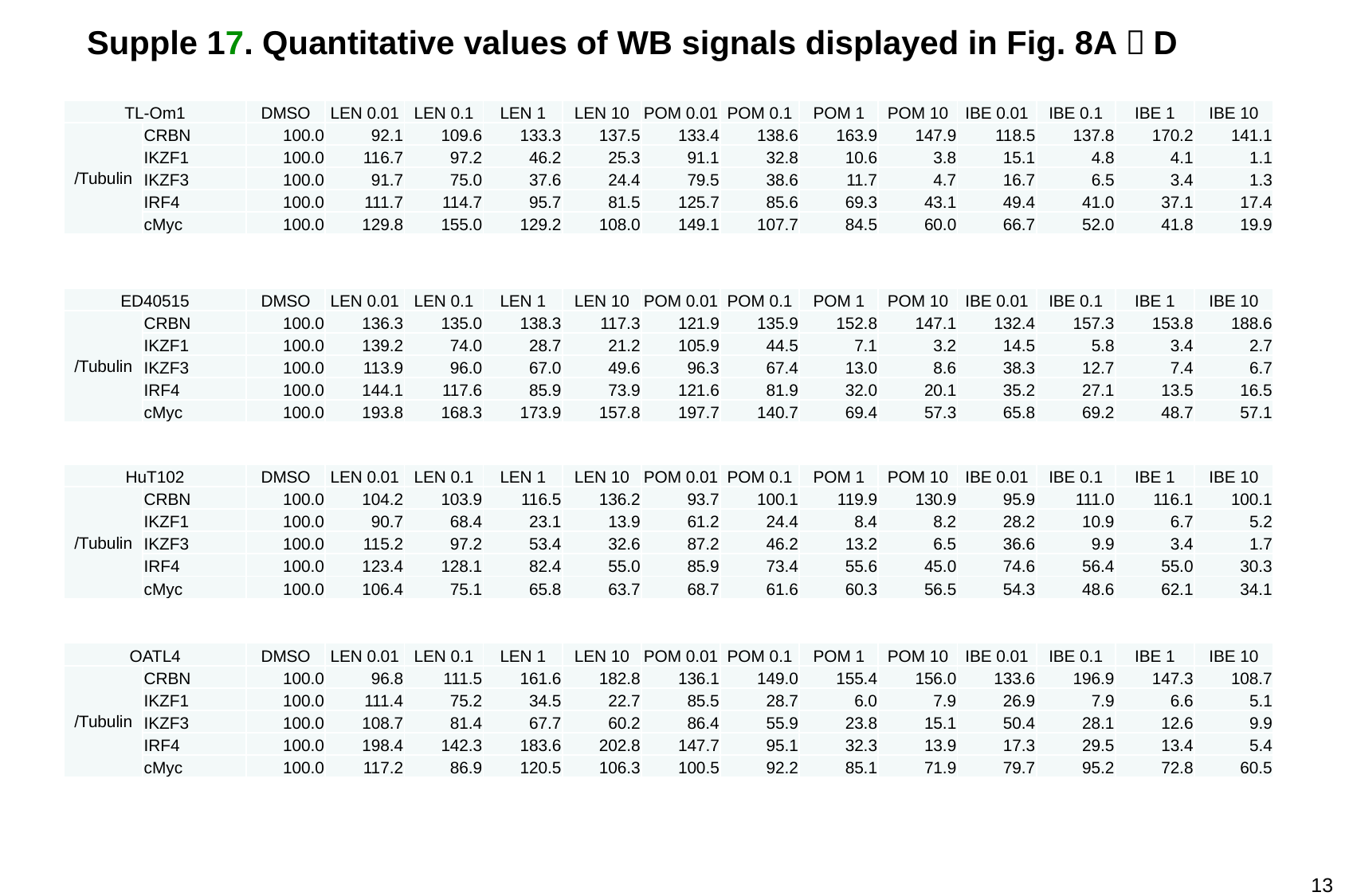

Supple 17. Quantitative values of WB signals displayed in Fig. 8A〜D
| TL-Om1 | | DMSO | LEN 0.01 | LEN 0.1 | LEN 1 | LEN 10 | POM 0.01 | POM 0.1 | POM 1 | POM 10 | IBE 0.01 | IBE 0.1 | IBE 1 | IBE 10 |
| --- | --- | --- | --- | --- | --- | --- | --- | --- | --- | --- | --- | --- | --- | --- |
| /Tubulin | CRBN | 100.0 | 92.1 | 109.6 | 133.3 | 137.5 | 133.4 | 138.6 | 163.9 | 147.9 | 118.5 | 137.8 | 170.2 | 141.1 |
| | IKZF1 | 100.0 | 116.7 | 97.2 | 46.2 | 25.3 | 91.1 | 32.8 | 10.6 | 3.8 | 15.1 | 4.8 | 4.1 | 1.1 |
| | IKZF3 | 100.0 | 91.7 | 75.0 | 37.6 | 24.4 | 79.5 | 38.6 | 11.7 | 4.7 | 16.7 | 6.5 | 3.4 | 1.3 |
| | IRF4 | 100.0 | 111.7 | 114.7 | 95.7 | 81.5 | 125.7 | 85.6 | 69.3 | 43.1 | 49.4 | 41.0 | 37.1 | 17.4 |
| | cMyc | 100.0 | 129.8 | 155.0 | 129.2 | 108.0 | 149.1 | 107.7 | 84.5 | 60.0 | 66.7 | 52.0 | 41.8 | 19.9 |
| ED40515 | | DMSO | LEN 0.01 | LEN 0.1 | LEN 1 | LEN 10 | POM 0.01 | POM 0.1 | POM 1 | POM 10 | IBE 0.01 | IBE 0.1 | IBE 1 | IBE 10 |
| --- | --- | --- | --- | --- | --- | --- | --- | --- | --- | --- | --- | --- | --- | --- |
| /Tubulin | CRBN | 100.0 | 136.3 | 135.0 | 138.3 | 117.3 | 121.9 | 135.9 | 152.8 | 147.1 | 132.4 | 157.3 | 153.8 | 188.6 |
| | IKZF1 | 100.0 | 139.2 | 74.0 | 28.7 | 21.2 | 105.9 | 44.5 | 7.1 | 3.2 | 14.5 | 5.8 | 3.4 | 2.7 |
| | IKZF3 | 100.0 | 113.9 | 96.0 | 67.0 | 49.6 | 96.3 | 67.4 | 13.0 | 8.6 | 38.3 | 12.7 | 7.4 | 6.7 |
| | IRF4 | 100.0 | 144.1 | 117.6 | 85.9 | 73.9 | 121.6 | 81.9 | 32.0 | 20.1 | 35.2 | 27.1 | 13.5 | 16.5 |
| | cMyc | 100.0 | 193.8 | 168.3 | 173.9 | 157.8 | 197.7 | 140.7 | 69.4 | 57.3 | 65.8 | 69.2 | 48.7 | 57.1 |
| HuT102 | | DMSO | LEN 0.01 | LEN 0.1 | LEN 1 | LEN 10 | POM 0.01 | POM 0.1 | POM 1 | POM 10 | IBE 0.01 | IBE 0.1 | IBE 1 | IBE 10 |
| --- | --- | --- | --- | --- | --- | --- | --- | --- | --- | --- | --- | --- | --- | --- |
| /Tubulin | CRBN | 100.0 | 104.2 | 103.9 | 116.5 | 136.2 | 93.7 | 100.1 | 119.9 | 130.9 | 95.9 | 111.0 | 116.1 | 100.1 |
| | IKZF1 | 100.0 | 90.7 | 68.4 | 23.1 | 13.9 | 61.2 | 24.4 | 8.4 | 8.2 | 28.2 | 10.9 | 6.7 | 5.2 |
| | IKZF3 | 100.0 | 115.2 | 97.2 | 53.4 | 32.6 | 87.2 | 46.2 | 13.2 | 6.5 | 36.6 | 9.9 | 3.4 | 1.7 |
| | IRF4 | 100.0 | 123.4 | 128.1 | 82.4 | 55.0 | 85.9 | 73.4 | 55.6 | 45.0 | 74.6 | 56.4 | 55.0 | 30.3 |
| | cMyc | 100.0 | 106.4 | 75.1 | 65.8 | 63.7 | 68.7 | 61.6 | 60.3 | 56.5 | 54.3 | 48.6 | 62.1 | 34.1 |
| OATL4 | | DMSO | LEN 0.01 | LEN 0.1 | LEN 1 | LEN 10 | POM 0.01 | POM 0.1 | POM 1 | POM 10 | IBE 0.01 | IBE 0.1 | IBE 1 | IBE 10 |
| --- | --- | --- | --- | --- | --- | --- | --- | --- | --- | --- | --- | --- | --- | --- |
| /Tubulin | CRBN | 100.0 | 96.8 | 111.5 | 161.6 | 182.8 | 136.1 | 149.0 | 155.4 | 156.0 | 133.6 | 196.9 | 147.3 | 108.7 |
| | IKZF1 | #DIV/0! | #DIV/0! | #DIV/0! | #DIV/0! | #DIV/0! | #DIV/0! | #DIV/0! | #DIV/0! | #DIV/0! | #DIV/0! | #DIV/0! | #DIV/0! | #DIV/0! |
| | IKZF3 | 100.0 | 108.7 | 81.4 | 67.7 | 60.2 | 86.4 | 55.9 | 23.8 | 15.1 | 50.4 | 28.1 | 12.6 | 9.9 |
| | IRF4 | 100.0 | 198.4 | 142.3 | 183.6 | 202.8 | 147.7 | 95.1 | 32.3 | 13.9 | 17.3 | 29.5 | 13.4 | 5.4 |
| | cMyc | 100.0 | 117.2 | 86.9 | 120.5 | 106.3 | 100.5 | 92.2 | 85.1 | 71.9 | 79.7 | 95.2 | 72.8 | 60.5 |
| OATL4 | | DMSO | LEN 0.01 | LEN 0.1 | LEN 1 | LEN 10 | POM 0.01 | POM 0.1 | POM 1 | POM 10 | IBE 0.01 | IBE 0.1 | IBE 1 | IBE 10 |
| --- | --- | --- | --- | --- | --- | --- | --- | --- | --- | --- | --- | --- | --- | --- |
| /Tubulin | CRBN | 100.0 | 96.8 | 111.5 | 161.6 | 182.8 | 136.1 | 149.0 | 155.4 | 156.0 | 133.6 | 196.9 | 147.3 | 108.7 |
| | IKZF1 | 100.0 | 111.4 | 75.2 | 34.5 | 22.7 | 85.5 | 28.7 | 6.0 | 7.9 | 26.9 | 7.9 | 6.6 | 5.1 |
| | IKZF3 | 100.0 | 108.7 | 81.4 | 67.7 | 60.2 | 86.4 | 55.9 | 23.8 | 15.1 | 50.4 | 28.1 | 12.6 | 9.9 |
| | IRF4 | 100.0 | 198.4 | 142.3 | 183.6 | 202.8 | 147.7 | 95.1 | 32.3 | 13.9 | 17.3 | 29.5 | 13.4 | 5.4 |
| | cMyc | 100.0 | 117.2 | 86.9 | 120.5 | 106.3 | 100.5 | 92.2 | 85.1 | 71.9 | 79.7 | 95.2 | 72.8 | 60.5 |
13

## Slide 14
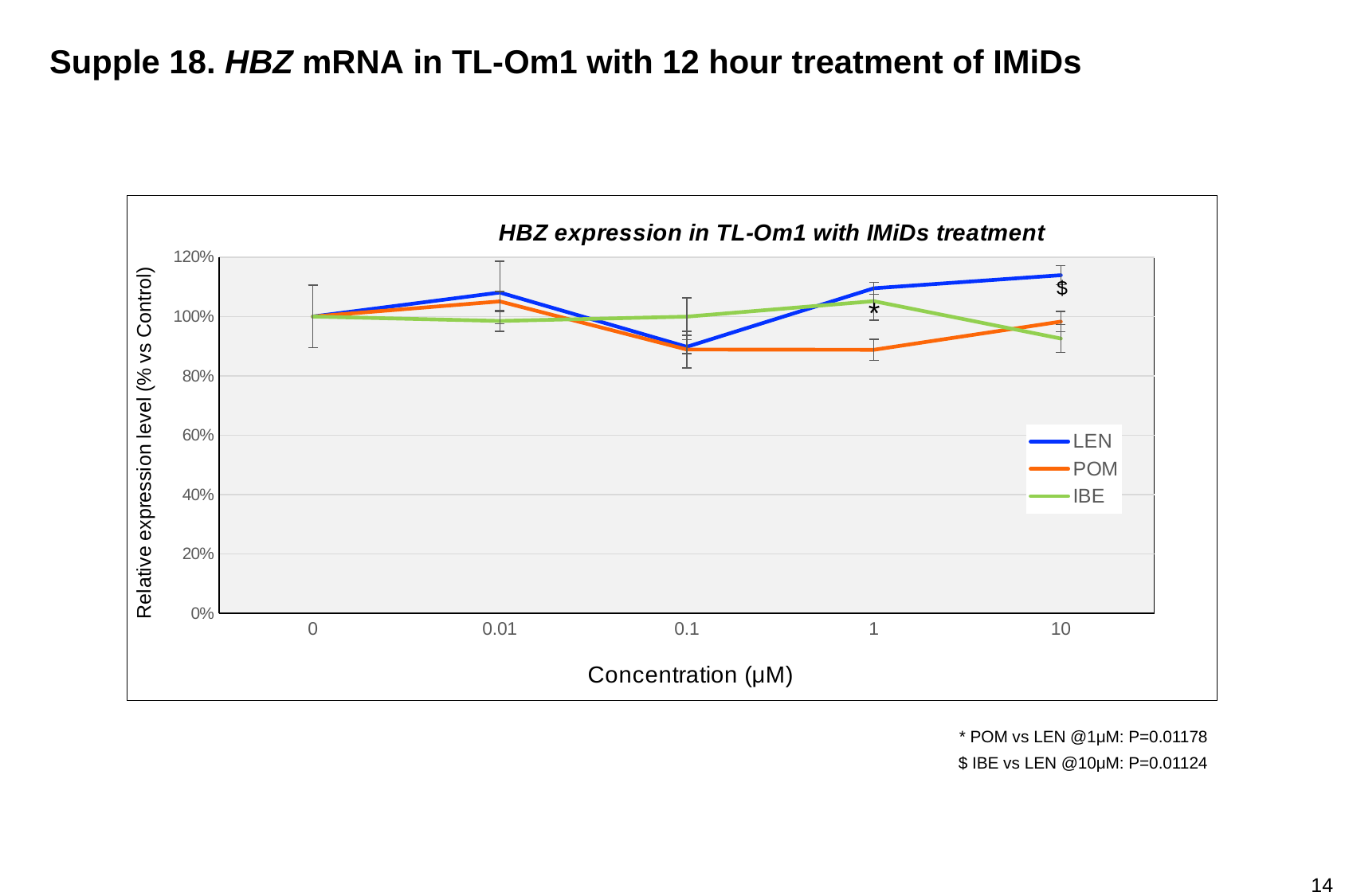

Supple 18. HBZ mRNA in TL-Om1 with 12 hour treatment of IMiDs
### Chart: HBZ expression in TL-Om1 with IMiDs treatment
| Category | LEN | POM | IBE |
|---|---|---|---|
| 0 | 1.0 | 1.0 | 1.0 |
| 0.01 | 1.08103473982406 | 1.05099150141643 | 0.98516475324288 |
| 0.1 | 0.898091546145818 | 0.888996570746981 | 0.999776353063963 |
| 1 | 1.09527359475175 | 0.888027434024154 | 1.05188608916058 |
| 10 | 1.13933204115104 | 0.982928283882511 | 0.925972864171761 |$
*
| \* POM vs LEN @1μM: P=0.01178 |
| --- |
| $ IBE vs LEN @10μM: P=0.01124 |
14

## Slide 15
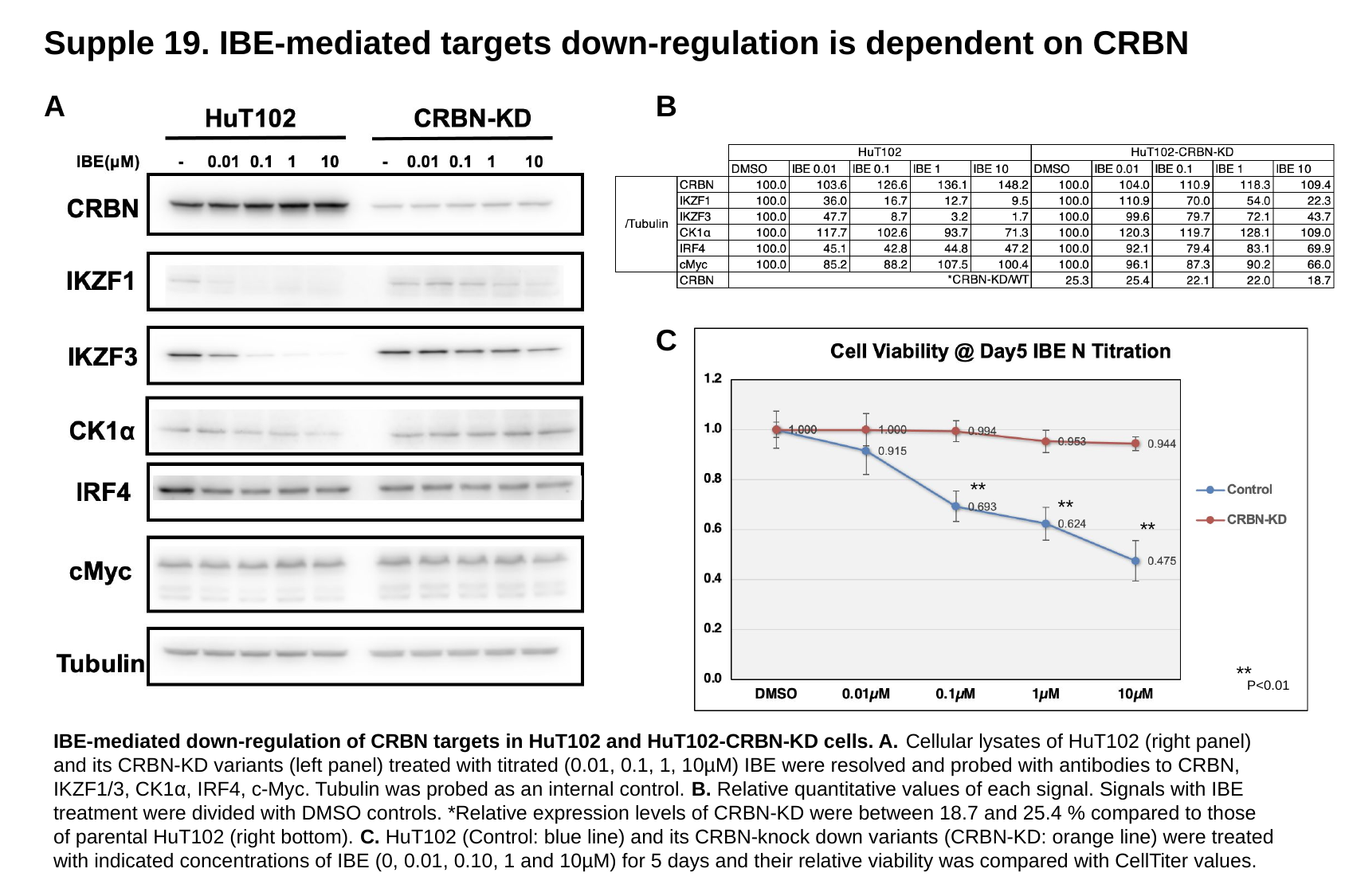

Supple 19. IBE-mediated targets down-regulation is dependent on CRBN
A
B
C
**
**
**
**
P<0.01
IBE-mediated down-regulation of CRBN targets in HuT102 and HuT102-CRBN-KD cells. A. Cellular lysates of HuT102 (right panel)
and its CRBN-KD variants (left panel) treated with titrated (0.01, 0.1, 1, 10µM) IBE were resolved and probed with antibodies to CRBN,
IKZF1/3, CK1α, IRF4, c-Myc. Tubulin was probed as an internal control. B. Relative quantitative values of each signal. Signals with IBE
treatment were divided with DMSO controls. *Relative expression levels of CRBN-KD were between 18.7 and 25.4 % compared to those
of parental HuT102 (right bottom). C. HuT102 (Control: blue line) and its CRBN-knock down variants (CRBN-KD: orange line) were treated with indicated concentrations of IBE (0, 0.01, 0.10, 1 and 10µM) for 5 days and their relative viability was compared with CellTiter values.
